# The genetic association between bipolar disorder and dementia: a qualitative review

**DOI:** 10.3389/fpsyt.2024.1414776

**Published:** 2024-08-20

**Authors:** Hirofumi Hirakawa, Takeshi Terao

**Affiliations:** Department of Neuropsychiatry, Oita University Faculty of Medicine, Yufu, Oita, Japan

**Keywords:** bipolar disorder, dementia, gene, single nucleotide polymorphisms, genome-wide association study

## Abstract

Bipolar disorder is a chronic disorder characterized by fluctuations in mood state and energy and recurrent episodes of mania/hypomania and depression. Bipolar disorder may be regarded as a neuro-progressive disorder in which repeated mood episodes may lead to cognitive decline and dementia development. In the current review, we employed genome-wide association studies to comprehensively investigate the genetic variants associated with bipolar disorder and dementia. Thirty-nine published manuscripts were identified: 20 on bipolar disorder and 19 on dementia. The results showed that the genes CACNA1C, GABBR2, SCN2A, CTSH, MSRA, and SH3PXD2A were overlapping between patients with bipolar disorder and dementia. In conclusion, the genes CACNA1C, GABBR2, SCN2A, CTSH, MSRA, and SH3PXD2A may be associated with the neuro-progression of bipolar disorder to dementia. Further genetic studies are needed to comprehensively clarify the role of genes in cognitive decline and the development of dementia in patients with bipolar disorder.

## Introduction

Bipolar disorder is a chronic disorder characterized by fluctuations in mood state and energy, in addition to recurrent episodes of mania/hypomania and depression (1). Cognitive impairment has been documented in a variety of neuropsychological domains during the mood disturbances associated with the acute episodes of bipolar disorder (2, 3). However, patients with bipolar disorder suffer from cognitive impairment not only during the acute phase but also during the remission phase (4, 5). Bipolar disorder in the first episode is associated with widespread cognitive dysfunction, especially in psychomotor speed, attention, working memory, and cognitive flexibility, suggesting that a broad range of cognitive deficits is already present at this early stage (6). Cognitive decline occurs with repeated manic episodes, hospitalizations, and length of illness in patients with bipolar disorder, suggesting that the recurrence of mania may have a long-term neuropsychological impact (7, 8). Studies on bipolar disorder have shown that neuropsychological deficits are detectable in euthymia, and they contribute to poor outcomes (8, 9). Patients with bipolar disorder have the greatest risk of dementia, followed by those with unipolar depression, schizophrenia, and neurosis (10). A meta-analysis revealed that a history of bipolar disorder significantly increased the risk of dementia (odds ratio: 2.36; 95% CI: 1.36–4.09) (11). Another meta-analysis showed that bipolar disorder increases the risk of dementia (odds ratio: 2.96; 95% CI: 2.09–4.18), and the risk of progression to dementia is higher in bipolar disorder than that in major depressive disorder (12). Thus, bipolar disorder may be seen as a neuro-progressive disorder in which repeated mood episodes may lead to cognitive decline and, finally, the development of dementia.

Genome-wide association studies (GWAS) aim to identify single nucleotide polymorphisms (SNPs) in which allele frequencies vary systematically as a function of phenotypic trait values (13). The identification of trait-associated SNPs distributed throughout the genome may provide new insights into the biological mechanisms underlying psychiatric disorders (13). To date, GWAS have successfully identified SNPs associated with the risk of bipolar disorder and dementia (14, 15). The clinical question arises as to which genetic factors are associated with the association between bipolar disorder and dementia. From a clinical perspective, we previously hypothesized that there is a specific group of patients whose diagnoses longitudinally change from depression to bipolar disorder and finally to dementia, and the glycogen synthase kinase 3β gene may be a common etiological factor in these diseases and diagnostic conversions (16). In the current review, using a completely different perspective, we employed the results of GWAS to comprehensively investigate genetic variants associated with bipolar disorder and dementia (including Alzheimer’s disease, Lewy body dementia, frontotemporal dementia, and vascular dementia).

## Methods

This review was qualitative and not systematic in nature. This study was conducted in January, 2024. Using the PubMed database, we conducted searches with keywords “bipolar disorder” and “GWAS”, “dementia” and “GWAS”. In this review, only GWAS that examined the relationship between the diagnosis of bipolar disorder or dementia and genes and SNPs were included. Articles that were not GWAS or were written in languages other than English were excluded. We simply examined the presence of overlapping genes and SNPs reported in patients with bipolar disorder and dementia.

## Results

Thirty-nine published manuscripts were identified: 20 on bipolar disorder (14, 17–35) and 19 on dementia (15, 36–53) (see **Supplementary Figure 1** for the literature screening flow chart). **Table 1** summarizes the characteristics of the included studies. The lists of significant SNPs and genes associated with bipolar disorder and dementia reported by GWAS are provided in **Supplementary Tables 1**, **2**, respectively.

**Table 1:** The characteristics of the studies included in the present review.

Author	Diagnosis	Criteria	Sample size	Platform	GWAS software
Mullins et al., 2021 (14)	Bipolar disorder	DSM-IV, ICD-9, ICD-10	Bipolar disorder: 41,917, Controls:371,549	Affymetrix and Illumina	PLINK ver 1.90
Li et al., 2021 (17)	Bipolar disorder	DSM-IV	Bipolar disorder: 1784, Controls:2474	SNaPShot	PLINK ver 1.9
Budde et al., 2019 (18)	Bipolar disorder	DSM-IV	Bipolar disorder:1,081	Illumina; HumanHap550v3, Human610, Human660w	PLINK
Stahl et al., 2019 (19)	Bipolar disorder	DSM-IV or ICD-10	Bipolar disorder:20,352, Controls:31,358	Affymetrix and Illumina	PLINK ver 1.09
Ikeda et al., 2018 (20)	Bipolar disorder	DSM-IV-TR	Bipolar disorder: 2,964, Controls:61,887	Illumina; HumanOmniExpressExome v1.0/v1.2 chip	LocusZoom
Acikel et al., 2016 (21)	Bipolar disorder	–	Bipolar disorder: 604, Controls: 1,767	Affymetrix; Affy6.0	PLINK ver 1.8
Hou et al., 2016 (22)	Bipolar disorder	DSM-III or DSM-IV	Bipolar disorder: 9784, Controls:30,471	Affymetrix; Affy6.0, 500K and Illumina; HumanHap550, HumanOmni2.5M, OmniExpress	PLINK
Li et al., 2016 (23)	Bipolar disorder	DSM-IV, ICD-10	Bipolar disorder: 7,481, Controls: 9,250	Affymetrix exon arrays	Sherlock
Kuo et al., 2014 (24)	Bipolar disorder	DSM-IV	Bipolar disorder: 240, Controls: 240	Affymetrix; Affy6.0	PLINK ver 1.07
Mühleisen et al., 2014 (25)	Bipolar disorder	DSM-IIR, DSM-IV	Bipolar disorder: 9,747, Controls:14,278	Illumina; Human660W, HumanOmni1	PLINK ver 1.07, INTERSNP ver 1.11
Xu et al., 2014 (26)	Bipolar disorder	DSM-IV or ICD-10	Bipolar disorder: 950, Controls: 950	Affymetrix; Affy5.0	PLINK
Chen et al., 2013 (27)	Bipolar disorder	DSM-IV	Bipolar disorder: 7773, Controls:10 915	Affymetrix; Affy6.0, 500K and Illumina; HumanHap550, HumanHap300, human 610-Quad, Infinium II	PLINK ver 1.4
Green et al., 2013 (28)	Bipolar disorder	DSM-IV	Bipolar disorder: 7,481, Controls: 9,250	Illumina	PLINK ver 1.07
Cichon et al., 2011 (29)	Bipolar disorder	DSM-IV	Bipolar disorder: 682, Controls:1,300	Illumina; HumanHap550v3	PLINK ver 1.05
Lee et al., 2011 (30)	Bipolar disorder	DSM-IV	Bipolar disorder: 1409, Controls: 2000	Illumina; HumanHap550	–
Psychiatric GWAS Consortium Bipolar Disorder Working Group, 2011 (31)	Bipolar disorder	DSM-IV, ICD-10	Bipolar disorder: 11,974, Controls: 51,792	Affymetrix; Affy6.0, Affy5.0, 500K and Illumina; HumanHap550	PLINK
Yosifova et al., 2011 (32)	Bipolar disorder	–	Bipolar disorder: 188, Controls: 376	Illumina; HumanHap550v3	–
Djurovic et al., 2010 (33)	Bipolar disorder	DSM-IV	Bipolar disorder: 194, Controls:336	Affymetrix; Affy6.0 and Illumina; HumanHap300 and HumanCNV370	PLINK
Scott et al., 2009 (34)	Bipolar disorder	DSM-IV or ICD-10	Bipolar disorder: 3,683, Controls:14,507	Affymetrix; 500K and Illumina; HumanHap550	–
Ferreira et al., 2008 (35)	Bipolar disorder	DSM-IV	Bipolar disorder: 4,387, Controls: 6,209	Affymetrix; Affy6.0, Affy5.0, 500K	PLINK
Dalmasso et al., 2024 (36)	Alzheimer’s disease	NINCDS-ADRDA	Alzheimer’s disease: 539, Controls: 854	Illumina; Infinium Global Screening Array v.1.0	PLINK ver 1.9
Sherva et al., 2023 (37)	Alzheimer’s disease	ICD-9, ICD-10	Alzheimer’s disease: 4,012, Controls: 18,435	MVP 1.0 custom Axiom array	SHAPEIT4 v 4.1.3, MINIMAC4
Bellenguez et al., 2022 (15)	Alzheimer’s disease	NINCDS–ADRDA	Alzheimer’s disease:111,326, Controls:677,663	Affymetrix; 500K and Illumina; Human Omni Express-24 v1.1, Human 610, HumanHap550	SNPTEST 2.5.4-beta3, PLINK v1.90
Harper et al., 2022 (38)	Alzheimer’s disease	–	Alzheimer’s disease: 424, Controls: 2,206	Illumina;Infinium Multi-Ethnic Global-8 v1.0	PLINK
Wightman et al., 2021 (39)	Alzheimer’s disease	NINCDS–ADRDA, ICD-9, ICD-10	Alzheimer’s disease: 90,338, Controls:1,036,225	Illumina; HumanHap550, Human Omni Express, Infinium Global Screening Array	PLINK ver 1.90
Jansen et al., 2019 (40)	Alzheimer’s disease	NINCDS–ADRDA, ICD-10	Alzheimer’s disease:71,880, Controls:383,378	Affymetrix;UK BiLEVE, Illumina; Human Omni Express-24 v.1.1 Axiom array, UK Biobank Axiom array	PLINK
Kunkle et al., 2019 (41)	Alzheimer’s disease	NINCDS-ADRDA, DSM-IV, DSM-V	Alzheimer’s disease: 35,274, Controls:59,163	Affymetrix; 500K and Illumina; HumanHap550, 370CNV Duo, Human 610, OmniExpress	PLINK
Moreno-Grau et al., 2019 (42)	Alzheimer’s disease	–	Alzheimer’s disease: 11,999, Controls: 9,236	Affymetrix	PLINK ver 1.9
Witoelar et al., 2018 (43)	Alzheimer’s disease	ICD-10	Alzheimer’s disease: 2,135, Controls:6,858	Illumina; Human Omni Express-24 v.1.1	PLINK ver 1.9
Jun et al., 2016 (44)	Alzheimer’s disease	NINCDS-ADRDA	Alzheimer’s disease: 17,536, Controls: 3,6175	Affymetrix; Affy6.0, Illumina; Human Omni Express-24	–
Lambert et al., 2013 (45)	Alzheimer’s disease	NINCDS-ADRDA	Alzheimer’s disease: 17,008, Controls:37,154	Illumina; HumanHap550, 370CNV Duo, Human 610, Omni1	PLINK, probABEL, R (GEE), SNPtest
Seshadri et al., 2010 (46)	Alzheimer’s disease	NINCDS-ADRDA/DSM-IV	Alzheimer’s disease: 3,006, Controls:14,642	Illumina; HumanHap550, 370CNV Duo, Human 610	PLINK
Mukherjee et al., 2020 (47)	Late-onset Alzheimer’s disease	NINCDS–ADRDA	Late-onset Alzheimer’s disease: 2431, Controls:3447	–	PLINK ver 1.9
Beecham et al., 2009 (48)	Late-onset Alzheimer’s disease	NINCDS–ADRDA	Late-onset Alzheimer’s disease: 492, Controls:496	Illumina; HumanHap550	PLINK ver 1.9
Chia et al., 2021 (49)	Lewy body dementia	Established consensus criteria of DLB Consortium	Lewy body dementia: 2591, Controls:4027	Illumina; HiSeq X Ten sequencer	PLINK ver 1.9
Schrijvers et al., 2012 (50)	Vascular dementia	NINDS-AIREN	Vascular dementia: 67, Controls:5633	Illumina; HumanHap550 v3.0	PLINK
Reus et al., 2021 (51)	Frontotemporal dementia	Neary criteria, Rascovsky and Gorno-Tempini criteria	Frontotemporal dementia: 354, Controls: 4,209	Illumina; Genome Screening Array	PLINK ver 2.0
Ferrari et al., 2015 (52)	Frontotemporal dementia	Neary criteria, Rascovsky and Gorno-Tempini criteria	Frontotemporal dementia: 530, Controls:926	Illumina; Human 660K	PLINK
Ferrari et al., 2014 (53)	Frontotemporal dementia	Neary criteria	Frontotemporal dementia: 3526, Controls:9402	Illumina; Human 370K, 550K, 660K	PLINK ver 1.07

DSM, Diagnostic and Statistical Manual of Mental Disorders; ICD, International Classification of Diseases; NINCDS-ADRDA, National Institute of Neurological and Communicative Disorders and Stroke and the Alzheimer’s Disease and Related Disorders Association; NINDS-AIREN, Neurological Disorders and Stroke-Association Internationale pour la Recherche et l'Enseignement en Neurosciences.

A review revealed that among the genes reported to be associated with the diagnosis of bipolar disorder or dementia in previous GWAS studies, the overlapping gene was calcium voltage-gated channel subunit Alpha1 C (CACNA1C), gamma-aminobutyric acid B receptor 2 (GABBR2), sodium voltage-gated channel Alpha subunit 2 (SCN2A), cathepsin H (CTSH), methionine sulfoxide reductase A (MSRA), and SH3 and PX domains 2A (SH3PXD2A) (**Table 2**; **Supplementary Figure 2**). With respect to the type of dementia, rs11062164 of CACNA1C, rs16916777 of GABBR2, and rs17738042 of SH3PXD2A are associated with vascular dementia; rs10184275 and rs2119067 of SCN2A are associated with late-onset Alzheimer’s disease; and, rs12592898 of CTSH and rs4607615 of MSRA are associated with Alzheimer’s disease. No SNPs were found to be reported as associated with both the diagnosis of bipolar disorder and dementia.

**Table 2:** The overlapping genes and single nucleotide polymorphisms with bipolar disorder and dementia.

Diagnosis	SNPs	Position	Minor Alleles	Allele Directions	Author
Gene: CACNA1C					
Bipolar disorder	rs1006737	chr12:2236129	A	+	Chen et al., 2013 (27); Ferreira et al., 2008 (35); Green et al., 2013 (28)
Bipolar disorder	rs1024582	chr12:2293080	A	+	Ferreira et al., 2008 (35)
Bipolar disorder	rs10744560	chr12:2277933	T	+	Stahl et al., 2019 (19)
Bipolar disorder	rs10848642	chr12:2222406	G	–	Chen et al., 2013 (27)
Bipolar disorder	rs11062170	chr12:2239678	C	+	Mullins et al., 2021 (14)
Bipolar disorder	rs4765913	chr12:2310730	A	+	Green et al., 2013 (28); Psychiatric GWAS Consortium Bipolar Disorder Working Group, 2011 (31)
Vascular dementia	rs11062164	chr12:2224486	A	+	Schrijvers et al., 2012 (50)
Gene: GABBR2					
Bipolar disorder	rs7864144	chr9:98643756	G	–	Xu et al., 2014 (26)
Vascular dementia	rs16916777	chr9:98518893	A	+	Schrijvers et al., 2012 (50)
Gene: SCN2A					
Bipolar disorder	rs17183814	chr2:165295879	G	+	Mullins et al., 2021 (14); Stahl et al., 2019 (19)
Late-onset Alzheimer’s disease	rs10184275	chr2:165271418	–	–	Beecham et al., 2009 (48)
Late-onset Alzheimer’s disease	rs2119067	chr2:165270773	–	–	Beecham et al., 2009 (48)
Gene: CTSH					
Bipolar disorder	rs16970287	chr15:78935915	G	+	Yosifova et al., 2011 (32)
Bipolar disorder	rs2289700	chr15:78932341	A	+	Yosifova et al., 2011 (32)
Alzheimer’s disease	rs12592898	chr15:78936857	A	–	Bellenguez et al., 2022 (15)
Gene: MSRA					
Bipolar disorder	rs3088186	chr8:10368845	T	+	Mullins et al., 2021 (14)
Alzheimer’s disease	rs4607615	chr8:10422116	C	–	Sherva et al., 2023 (37)
Gene: SH3PXD2A					
Bipolar disorder	rs2281587	chr10:103617592	C	+	Psychiatric GWAS Consortium Bipolar Disorder Working Group, 2011 (31)
Vascular dementia	rs17738042	chr10:103596274	A	+	Schrijvers et al., 2012 (50)

CACNA1C, Calcium voltage-gated channel subunit Alpha1 C; CTSH, Cathepsin H; GABBR2, Gamma-aminobutyric acid B receptor 2; GWAS, Genome-wide association studies; MSRA, Methionine sulfoxide reductase A; SH3PXD2A, SH3 and PX domains 2A; SNPs, Single nucleotide polymorphisms; SCN2A ,Sodium voltage-gated channel Alpha subunit 2.

## Discussion

According to GWAS, the CACNA1C, GABBR2, SCN2A, CTSH, MSRA, and SH3PXD2A genes were common between bipolar disorder and dementia.

CACNA1C encodes the alpha-1 subunit of the voltage-dependent L-type calcium channel expressed in the human brain (54), which regulates cellular calcium influx and is essential for normal brain development and plasticity (55). Variants of CACNA1C have been associated with bipolar disorder and several neuropsychiatric disorders, such as schizophrenia, major depressive disorder, autism spectrum disorder, attention deficit hyperactivity disorder, and substance-use disorders (56). The CACNA1C gene (especially the rs1006737 A allele) is robustly associated with bipolar disorder and might be crucial in molecular biological research on the set of interacting proteins involved in the calcium channel activity in bipolar disorder (57). The CACNA1C gene may not only be associated with the onset of bipolar disorder but also potentially affects the course of cognitive function and brain imaging. The rs1006737 variant (minor allele: A) of the CACNA1C gene is associated with cognitive impairment in patients with bipolar disorder and schizophrenia spectrum (58). Furthermore, a 2-year longitudinal study on bipolar disorder revealed that patients with the AA genotype of rs1006737 showed poorer cognitive performance, particularly in terms of processing speed (59). The rs10466907 variant of CACNA1C is associated with cognitive recovery after a major depressive episode in bipolar disorder (60). CACNA1C is expressed throughout the mouse brain, including key limbic regions relevant for emotion and cognition, such as the prefrontal cortex, hippocampus, and amygdala (61). Moreover, embryonic deletion of CACNA1C in glutamatergic neurons in the forebrain promotes the manifestation of endophenotypes related to psychiatric disorders, including cognitive decline, impaired synaptic plasticity, reduced sociability, hyperactivity, and increased anxiety (61). In a human brain study on bipolar disorder, the rs1006737 A allele of the CACNA1C gene was associated with gray matter volume, functional connectivity within the corticolimbic frontotemporal neural system, and mean thickness of cortical brain areas (62, 63). Patients with bipolar disorder carrying the rs1006737 A allele showed age-related cortical thinning of the left caudal anterior cingulate cortex (64). In contrast, among non-A carriers, age did not affect cortical thinning in the left caudal anterior cingulate cortex, suggesting an underlying relationship with aging-associated cognitive decline (64). Furthermore, tau phosphorylation is known to increase in the cerebrospinal fluid (CSF) in patients with Alzheimer’s disease (65), and the rs1006737 variant of CACNA1C is significantly associated with hyperphosphorylated tau/total tau ratio in the CSF of patients with bipolar disorder (66). A study on late-onset Alzheimer’s disease focusing on protein–protein network interactions revealed eight genes with strong associations: APOE, SORL1, APOC1, CD33, CLU, TOMM40, CNTNAP2, and CACNA1C (67). Interestingly, CACNA1C (rs10848683 variant) is also associated with ischemic stroke (68). Similarly, CACNA1C (rs11062164 variant) was significantly associated with vascular dementia (50). Thus, CACNA1C is associated with dementia. Furthermore, the rs7297582 T allele of CACNA1C is associated with a risk of bipolar disorder and poor cognitive performance (69). Although no studies have reported an association between rs7297582 and dementia, future studies should consider SNPs of interest in relation to both bipolar disorder and dementia. In the present review, the SNPs of CACNA1C did not match across studies. CACNA1C, especially rs1006737 and rs7297582, may be strongly associated with the onset of bipolar disorder, cognitive decline in bipolar disorder, and dementia.

Human gamma-aminobutyric acid type B receptor (GABABR) is a G protein-coupled receptor central to inhibitory neurotransmission in the brain (70). GABABR is assembled by the heterodimeric interaction of the intracellular C-terminal tails of the two subunits encoded by GABBR1 and GABBR2 (70). In patients with bipolar disorder, GABAergic hypofunction has been observed in the cerebellum (71). Additionally, a post-mortem study revealed reduced protein expressions of GABBR1 and GABBR2 in the cerebellum of patients with bipolar disorder (72). Dysfunction of the GABAergic system may cause cognitive impairment in humans (73). The reduction in GABAergic system components in the brain and lower GABA levels in the CSF of patients with Alzheimer’s disease suggest that the GABAergic system is vulnerable to Alzheimer’s disease pathology and should be considered a potential target for developing pharmacological strategies and novel Alzheimer’s disease biomarkers (74, 75). GABBR2 is also associated with bipolar disorder and dementia.

SCN2A encodes the voltage-gated sodium channel Nav1.2, a major central nervous system sodium channel that plays a role in the initiation and conduction of action potentials (76). SCN2A variants are associated with a range of disorders including autism spectrum disorder, developmental delay, seizures, and epileptic encephalopathy (77). Furthermore, SCN2A is associated with psychiatric disorders such as bipolar disorder and schizophrenia (14, 78). SCN2A contributes to excitability that facilitates synapse formation and development (79). A study on Alzheimer’s disease focusing on protein-protein network interactions revealed six hub genes: SCN2A, SNAP25, GRIN2A, GRIN2B, DLG2, and ATP2B2 (80). Thus, SCN2A is associated with bipolar disorder and dementia.

CTSH is a cysteine cathepsin that primarily acts as an aminopeptidase (81). The main function of cathepsins is to degrade proteins via proteolysis in lysosomes (81). CTSH has been implicated in the cis-regulated mRNA association with Alzheimer’s disease (82). CTSH expression is significantly lower in the brain tissue of healthy controls than in patients with Alzheimer’s disease (83). Moreover, CTSH knockout affected genes related to endocytosis and significantly increased Aβ42 phagocytosis in microglial cells (83). The CTSH gene was significantly associated with Alzheimer’s disease (83). The mechanism by which CTSH is associated with bipolar disorder is unknown; however, a GWAS conducted by Yosifova et al. in a Bulgarian population identified a significant association between bipolar disorder and CTSH (32). Therefore, CTSH is a gene of interest related to bipolar disorder and dementia.

MSRA has been postulated to act as a catalytic antioxidant system that protects against oxidative stress-induced cell injury and is highly expressed in the brain (84, 85). The methionine sulfoxide reductase system may contribute to the development of aging-associated diseases, including neurodegenerative diseases (86). MRSA knockout mice exhibit enhanced neurodegeneration in the brain hippocampus compared to their wild-type counterparts (86). There are hypotheses suggesting that oxidative stress is associated with bipolar disorder and Alzheimer’s disease (87, 88). The rs4840463 polymorphism in MRSA is associated with an increased risk of bipolar I and executive function defects (87). In this review, the rs3088186 polymorphism is associated with bipolar disorder, while rs4607615 is linked to Alzheimer’s disease. Thus, MRSA is implicated in both bipolar disorder and dementia.

The SH3PXD2A gene encodes TKS5, an isoform essential for proper mammalian development (89). Additionally, SH3PXD2A directly interacts with the ADAM metallopeptidase domain 15 gene, which is involved in neurodegeneration and inflammatory processes (90). SH3PXD2A is associated with brain white matter lesions and stroke (90–92). It is noteworthy that SH3PXD2A (rs17738042) is significantly associated with vascular dementia (50). Interestingly, rs3740473 of SH3PXD2A is associated with Alzheimer’s disease (93). The mechanism by which SH3PXD2A is linked to bipolar disorder remains unknown; however, a GWAS revealed a significant association between bipolar disorder and SH3PXD2A (31). Therefore, SH3PXD2A is a gene of interest in relation to bipolar disorder and dementia.

In addition, we previously reviewed that rs334558 of the GSK-3β gene was associated with depression, bipolar disorder, and dementia (16, 94–96). We hypothesized the existence of a mental GSK-3 disease, which comprises a specific group of patients associated with the GSK-3β variant, whose diagnoses longitudinally transition from depression to bipolar disorder and finally to dementia (16). Therefore, although we could not find a significant association between the GSK-3β gene and bipolar disorder and dementia in this GWAS review, rs334558 of the GSK-3β gene is associated with bipolar disorder and dementia.

This review has several limitations. First, it relies on GWAS, which are cross-sectional in nature, and examines bipolar disorder and dementia at each study time point. Therefore, genetic studies involving the longitudinal transitions from bipolar disorder to dementia or cognitive function decline in patients with bipolar disorder are needed. Hence, future longitudinal studies are needed to explore the genetic factors associated with cognitive decline in bipolar disorder and the onset of dementia. Second, bipolar disorder and dementia are believed to be associated with various genes, and it is difficult to explain them based solely on a single gene or SNP. Therefore, it is necessary to consider factors from multiple genes, such as polygenic scores, which can summarize global genomic risk rather than focusing on specific variants. Third, this review only examined whether the identified genes or SNPs were relevant by extracting them from individual studies and did not involve a combined GWAS analysis of the cases in each study. Finally, this review is qualitative, not quantitative, and weighs the effects of candidate genes.

## Conclusion

In conclusion, CACNA1C, GABBR2, SCN2A, CTSH, MSRA, and SH3PXD2A may be associated with the neuro-progression of bipolar disorder to dementia. Further genetic studies are needed to comprehensively clarify the role of genes in cognitive decline and dementia development in patients with bipolar disorder.
