# Supplementary material for: The genetic association between bipolar disorder and dementia: a qualitative review
Source: Front Psychiatry. 2024 Aug 20;15:1414776. doi: 10.3389/fpsyt.2024.1414776 (PMC11368786; doi:10.3389/fpsyt.2024.1414776)
Supplement: Supplementary file 1 [file DataSheet1.docx]

Supplementary Material

**Supplementary Table 1: The List of significant single nucleotide polymorphism and genes of bipolar disorder by the genome-wide association study**

| Author | SNPs | Position | Minor Alleles | Allele Directions | Gene | Consequence |
| --- | --- | --- | --- | --- | --- | --- |
| Mullins et al, 2021 | rs2126180 | chr1:60639996 | A | + | LOC124904193 | Non Coding Transcript Variant |
|  | rs10737496 | chr1:163776152 | C | + | LOC124904447 | Intron Variant |
|  | rs4619651 | chr2:96750416 | G | + | None | None |
|  | rs17183814 | chr2:165295879 | G | + | SCN2A | Missense Variant |
|  | rs13417268 | chr2:168625327 | C | + | CERS6 | Intron Variant |
|  | rs2011302 | chr2:192873610 | A | + | None | None |
|  | rs2719164 | chr2:193573164 | A | + | None | None |
|  | rs9834970 | chr3:36814539 | C | + | None | None |
|  | rs2336147 | chr3:52592427 | T | + | PBRM1 | Intron Variant |
|  | rs115694474 | chr3:70439637 | T | + | None | None |
|  | rs696366 | chr3:108038213 | C | + | LOC124906265 | Non Coding Transcript Variant |
|  | rs112481526 | chr4:122154852 | G | + | KIAA1109 | Intron Variant |
|  | rs28565152 | chr5:7542798 | A | + | ADCY2 | Intron Variant |
|  | rs6865469 | chr5:79553682 | T | + | LOC105379047 | Intron Variant |
|  | rs6887473 | chr5:81665250 | G | + | SSBP2 | Intron Variant |
|  | rs10043984 | chr5:138376432 | T | + | KDM3B | Intron Variant |
|  | rs10866641 | chr5:169862202 | T | + | DOCK2, INSYN2B | Intron Variant, 3 Prime UTR Variant |
|  | rs13195402 | chr6:26463347 | G | + | BTN2A1 | Stop Gained |
|  | rs1487445 | chr6:98117335 | T | + | None | None |
|  | rs4331993 | chr6:152472437 | A | + | SYNE1 | Intron Variant |
|  | rs10455979 | chr6:166581772 | G | + | RPS6KA2 | Intron Variant |
|  | rs12668848 | chr7:1981360 | G | + | MAD1L1, LOC124901573 | Intron Variant, Intron Variant |
|  | rs113779084 | chr7:11832161 | A | + | THSD7A | 5 Prime UTR Variant |
|  | rs6954854 | chr7:21452971 | G | + | SP4 | Intron Variant |
|  | rs12672003 | chr7:24607603 | G | + | PALS2 | Intron Variant |
|  | rs11764361 | chr7:105402782 | A | + | None | None |
|  | rs6946056 | chr7:132185838 | C | + | PLXNA4 | Intron Variant |
|  | rs10255167 | chr7:140976353 | A | + | LOC105375536 | Intron Variant |
|  | rs62489493 | chr8:9906071 | G | + | LOC124902057 | Intron Variant |
|  | rs3088186 | chr8:10368845 | T | + | MSRA | Intron Variant |
|  | rs2953928 | chr8:34294974 | A | + | None | None |
|  | rs6992333 | chr8:143919209 | G | + | PLEC | Missense Variant |
|  | rs10973201 | chr9:37090541 | C | + | EBLN3P | 500B Downstream Variant |
|  | rs62581014 | chr9:138172038 | T | + | TUBBP5 | Intron Variant |
|  | rs1998820 | chr10:18462174 | T | + | CACNB2 | Intron Variant |
|  | rs10994415 | chr10:60562276 | C | + | ANK3 | Intron Variant |
|  | rs10761661 | chr10:62765375 | T | + | None | None |
|  | rs2273738 | chr10:109888901 | T | + | XPNPEP1 | Intron Variant |
|  | rs174592 | chr11:61851136 | G | + | FADS2 | Intron Variant |
|  | rs4672 | chr11:64242407 | A | + | FKBP2, LOC114841035 | Missense Variant, 3 Prime UTR Variant |
|  | rs475805 | chr11:66081267 | A | + | PACS1 | Intron Variant |
|  | rs678397 | chr11:66557112 | T | + | ACTN3 | Intron Variant |
|  | rs12575685 | chr11:70671822 | A | + | SHANK2 | Intron Variant |
|  | rs12289486 | chr11:79381483 | T | + | TENM4 | Intron Variant |
|  | rs11062170 | chr12:2239678 | C | + | CACNA1C | Intron Variant |
|  | rs35306827 | chr13:113214731 | G | + | CUL4A | Intron Variant |
|  | rs2693698 | chr14:99252882 | G | + | BCL11B | Intron Variant |
|  | rs35958438 | chr15:38681592 | G | + | LOC105370775 | Intron Variant |
|  | rs4447398 | chr15:42612706 | A | + | STARD9 | Intron Variant |
|  | rs62011709 | chr15:82863022 | T | + | HOMER2 | Intron Variant |
|  | rs748455 | chr15:84606344 | T | + | ZSCAN2, LOC105370947 | Intron Variant, Intron Variant |
|  | rs4702 | chr15:90883330 | G | + | FURIN, FES | Non Coding Transcript Variant, 2KB Upstream Variant |
|  | rs28455634 | chr16:9136959 | G | + | None | None |
|  | rs7199910 | chr16:9832491 | G | + | GRIN2A | Intron Variant |
|  | rs12932628 | chr16:89566317 | T | + | RPL13 | 3 Prime UTR Variant |
|  | rs4790841 | chr17:1932188 | T | + | None | None |
|  | rs11870683 | chr17:39973588 | T | + | GSDMA | Intron Variant |
|  | rs61554907 | chr17:40064179 | T | + | THRA | Intron Variant |
|  | rs228768 | chr17:44114525 | G | + | HDAC5, LOC105371789 | Intron Variant, 2KB Upstream Variant |
|  | rs67712855 | chr20:45053910 | T | + | STK4 | Intron Variant |
|  | rs6032110 | chr20:45315683 | A | + | RBPJL | Intron Variant |
|  | rs237460 | chr20:49416590 | T | + | KCNB1, LOC105372649 | Intron Variant, Intron Variant |
|  | rs13044225 | chr20:62290759 | G | + | OSBPL2 | Intron Variant |
|  | rs5758064 | chr22:40757875 | T | + | None | None |
|  |  |  |  |  |  |  |
| Li et al, 2021 | rs9834970 | chr3:36814539 | C | + | None | None |
|  | rs4789 | chr3:36827948 | C | + | TRANK1 | 3 Prime UTR Variant |
|  |  |  |  |  |  |  |
| Budde et al, 2019 | rs12906122 | chr15:48965004 | T | + | SHC4 | 2KB Upstream Variant |
|  | rs4474633 | chr15:48968404 | A | + | None | None |
|  | rs11631087 | chr15:48969988 | G | + | None | None |
|  | rs7173509 | chr15:48979887 | T | - | None | None |
|  | rs8037958 | chr15:48987209 | A | + | None | None |
|  | rs11854184 | chr15:49000997 | A | - | SECISBP2L | Missense Variant |
|  | rs7167402 | chr15:49009096 | A | + | SECISBP2L | Intron Variant |
|  | rs4457942 | chr15:49016326 | T | + | SECISBP2L | Intron Variant |
|  | rs8029751 | chr15:49031658 | A | + | SECISBP2L | Intron Variant |
|  | rs4775798 | chr15:49037239 | A | + | SECISBP2L | Intron Variant |
|  | rs7177541 | chr15:49050433 | A | + | None | None |
|  | rs10519206 | chr15:49058222 | T | - | None | None |
|  | rs11070681 | chr15:49066320 | G | + | None | None |
|  | rs2413930 | chr15:49083018 | T | + | None | None |
|  | rs2413931 | chr15:49083128 | C | + | None | None |
|  | rs2413932 | chr15:49091284 | C | - | None | None |
|  | rs12148348 | chr15:49104667 | C | + | None | None |
|  | rs11633810 | chr15:49124549 | C | - | COPS2 | 3 Prime UTR Variant |
|  | rs3088333 | chr15:49140891 | T | - | COPS2 | Intron Variant |
|  | rs12915792 | chr15:49146129 | T | - | COPS2 | Intron Variant |
|  | rs2413935 | chr15:49158747 | C | - | GALK2, NDUFAF4P1 | Intron Variant, 500B Downstream Variant |
|  | rs586758 | chr15:49216375 | A | + | GALK2 | Intron Variant |
|  | rs17396612 | chr15:49257746 | G | - | GALK2 | Intron Variant |
|  | rs2086256 | chr15:49265829 | T | + | GALK2 | Intron Variant |
|  | rs1904317 | chr15:49270069 | T | + | GALK2 | Intron Variant |
|  | rs16962243 | chr15:49270535 | T | + | GALK2 | Intron Variant |
|  | rs11854557 | chr15:49280763 | C | - | GALK2 | Intron Variant |
|  | rs7177959 | chr15:49293782 | A | - | GALK2 | Intron Variant |
|  | rs11635005 | chr15:49309228 | T | - | GALK2 | Intron Variant |
|  | rs7179127 | chr15:49311418 | T | - | GALK2 | Intron Variant |
|  | rs2078024 | chr15:49329413 | G | - | GALK2, FAM227B | Non Coding Transcript Variant, Intron Variant |
|  | rs10851475 | chr15:49333733 | A | + | FAM227B, GALK2 | Intron Variant, Intron Variant |
|  | rs16962414 | chr15:49384692 | G | - | FAM227B | Intron Variant |
|  | rs16962418 | chr15:49392588 | T | - | FAM227B | Intron Variant |
|  | rs12591300 | chr15:49412544 | A | - | FAM227B | Intron Variant |
|  | rs1904316 | chr15:49416513 | C | - | FAM227B | Intron Variant |
|  | rs1429555 | chr15:49419900 | A | - | FAM227B | Intron Variant |
|  | rs12592277 | chr15:49436562 | A | - | FAM227B, FGF7 | Intron Variant, Intron Variant |
|  | rs4316697 | chr15:49437728 | A | + | FAM227B, FGF7 | Intron Variant, Intron Variant |
|  | rs7168316 | chr15:49441549 | T | - | FAM227B, FGF7 | Intron Variant, Intron Variant |
|  | rs4338740 | chr15:49443100 | C | - | FAM227B, FGF7 | Intron Variant, Intron Variant |
|  | rs11634375 | chr15:49457351 | T | - | FAM227B, FGF7 | Intron Variant, Intron Variant |
|  | rs11639111 | chr15:49457538 | T | - | FAM227B, FGF7 | Intron Variant, Intron Variant |
|  | rs4480740 | chr15:49463645 | A | - | FAM227B, FGF7 | Intron Variant, Intron Variant |
|  |  |  |  |  |  |  |
| Stahl al, 2019 | rs7544145 | chr1:150166501 | T | + | LINC02988 | Intron Variant |
|  | rs57195239 | chr2:96710670-96710671 | I | - | LMAN2L | Intron Variant |
|  | rs17183814 | chr2:165295879 | A | - | SCN2A | Missense Variant |
|  | rs61332983 | chr2:193600988-193600995 | I | - | None | None |
|  | rs9834970 | chr3:36814539 | T | - | None | None |
|  | rs2302417 | chr3:52780240 | A | - | ITIH1 | Intron Variant |
|  | rs3804640 | chr3:108074862 | A | + | CD47 | Intron Variant |
|  | rs11724116 | chr4:161372886 | T | - | None | None |
|  | rs200550695 | chr5:7587124-7587126 | I | - | ADCY2 | Intron Variant |
|  | rs10035291 | chr5:81500549 | T | + | SSBP2 | Intron Variant |
|  | rs57970360 | chr6:71809692-71809694 | D | + | None | None |
|  | rs2388334 | chr6:98143746 | A | - | None | None |
|  | rs10455979 | chr6:166581772 | C | - | RPS6KA2 | Intron Variant |
|  | rs113779084 | chr7:11832161 | A | + | THSD7A | 5 Prime UTR Variant |
|  | rs73188321 | chr7:105407711 | T | - | None | None |
|  | rs201231874 | chr7:141000207 | D | - | None | None |
|  | rs10994318 | chr10:60366098 | C | + | ANK3 | Intron Variant |
|  | rs59134449 | chr10:109985805-109985814 | I | + | ADD3-AS1 | Intron Variant |
|  | rs12226877 | chr11:61824435 | A | + | FADS2, LOC124902680 | Intron Variant, 500B Downstream Variant |
|  | rs10896090 | chr11:66177715 | A | + | PACS1 | Intron Variant |
|  | rs7122539 | chr11:66895260 | A | - | PC | Intron Variant |
|  | rs12575685 | chr11:70671822 | A | + | SHANK2 | Intron Variant |
|  | rs10744560 | chr12:2277933 | T | + | CACNA1C, CACNA1C-IT3 | Intron Variant, Intron Variant |
|  | rs4447398 | chr15:42612706 | A | + | STARD9 | Intron Variant |
|  | rs139221256 | chr15:84814627-84814635 | I | - | None | None |
|  | rs11647445 | chr16:9833109 | T | - | GRIN2A | Intron Variant |
|  | rs112114764 | chr17:44123673 | T | - | HDAC5 | 2KB Upstream Variant |
|  | rs11557713 | chr18:62576643 | A | + | ZCCHC2 | Non Coding Transcript Variant |
|  | rs111444407 | chr19:19247398 | T | + | NCAN | Intron Variant |
|  | rs202012857 | chr20:45053909 | I | - | STK4 | Intron Variant |
|  | rs55648125 | chr6:50849005 | A | - | None | None |
|  | rs17150022 | chr7:24732158 | T | - | GSDME | Intron Variant |
|  | rs138321 | chr22:40813300 | A | + | SLC25A17 | Intron Variant |
|  | rs884301 | chr17:55290103 | T | + | HLF | Intron Variant |
|  | rs329319 | chr5:134570919 | A | + | JADE2 | Intron Variant |
|  | rs73496688 | chr11:79445704 | A | + | None | None |
|  | rs57681866 | chr2:57748579 | A | - | None | None |
|  | rs13231398 | chr7:110557355 | C | - | LOC105375452 | Intron Variant |
|  |  |  |  |  |  |  |
| Ikeda et al, 2018 | rs4523096 | chr6:152472277 | A | + | SYNE1 | Intron Variant |
|  | rs4332037 | chr7:1911173 | T | + | MAD1L1 | Intron Variant |
|  | rs174576 | chr11:61836038 | A | + | FADS2 | Intron Variant |
|  | rs12576775 | chr11:79366149 | G | + | TENM4 | Intron Variant |
|  | rs10875914 | chr12:49028311 | G | + | KMT2D | Intron Variant |
|  | rs4926298 | chr19:13042221 | G | + | NFIX | Intron Variant |
|  | rs72874541 | chr3:63148173 | G | + | None | None |
|  | rs3931398 | chr7:1991957 | A | + | MAD1L1 | Intron Variant |
|  | rs78089757 | chr10:125424260 | A | + | None | None |
|  | rs28456 | chr11:61822009 | G | + | FADS2, LOC124902680 | Intron Variant, Intron Variant |
|  | rs329674 | chr11:133907053 | A | + | IGSF9B | 3 Prime UTR Variant |
|  | rs185880008 | chr12:38807668 | T | + | CPNE8 | Intron Variant |
|  | rs9834970 | chr3:36814539 | C | + | None | None |
|  | rs76317718 | chr17:8319459 | G | + | ARHGEF15 | Intron Variant |
|  |  |  |  |  |  |  |
| Acikel et al, 2016 | rs6785 | chr2:207603273 | - | - | CREB1, METTL21A | Non Coding Transcript Variant, Intron Variant |
|  | rs2194124 | chr4:77268903 | - | - | None | None |
|  | rs4792189 | chr17:11870443 | - | - | DNAH9 | Intron Variant |
|  | rs7569781 | chr2:224966869 | - | - | DOCK10 | Intron Variant |
|  | rs9375098 | chr6:98033916 | - | - | None | None |
|  | rs10415145 | chr19:32350147 | - | - | ZNF507, LOC124904689 | Intron Variant, 2KB Upstream Variant |
|  | rs10857580 | chr10:48478341 | - | - | ARHGAP22 | Intron Variant |
|  | rs11015814 | chr10:27485903 | - | - | None | None |
|  | rs11015877 | chr10:27570308 | - | - | LINC02680 | Intron Variant |
|  | rs732183 | chr1:99224503 | - | - | None | None |
|  | rs11023096 | chr11:2506773 | - | - | KCNQ1 | Intron Variant |
|  | rs1328392 | chr6:152777092 | - | - | None | None |
|  | rs2791142 | chr1:163494893 | - | - | None | None |
|  | rs1861226 | chr2:58425881 | - | - | None | None |
|  | rs4654814 | chr1:22767928 | - | - | EPHB2 | Intron Variant |
|  | rs219506 | chr2:21295746 | - | - | None | None |
|  | rs2055710 | chr2:224901446 | - | - | DOCK10 | Intron Variant |
|  | rs2483023 | chr10:36550309 | - | - | None | None |
|  | rs9372649 | chr6:97947329 | - | - | None | None |
|  | rs12145634 | chr1:205429436 | - | - | LEMD1 | Intron Variant |
|  | rs17736182 | chr13:69857470 | - | - | KLHL1 | Intron Variant |
|  |  |  |  |  |  |  |
| Hou et al, 2016 | rs9834970 | chr3:36814539 | T | - | None | None |
|  | rs1487441 | chr6:98106018 | A | + | None | None |
|  | rs4236274 | chr7:1856777 | G | - | MAD1L1 | Intron Variant |
|  | rs12553324 | chr9:23347867 | G | + | None | None |
|  | rs1054442 | chr12:48995537 | C | + | DDN | 3 Prime UTR Variant |
|  | rs2517959 | chr17:39690259 | A | + | ERBB2 | Intron Variant |
|  |  |  |  |  |  |  |
| Li et al, 2016 | rs2251219 | chr3:52550771 | - | - | PBRM1, LOC124909379 | Synonymous Variant, Intron Variant |
|  | rs12634640 | chr3:186351776 | - | - | DGKG | Intron Variant |
|  | rs810517 | chr10:79182863 | - | - | ZMIZ1 | Intron Variant |
|  | rs10501340 | chr11:55915319 | - | - | OR5W2 | 2KB Upstream Variant |
|  | rs6088662 | chr20:34959830 | - | - | MYH7B | Intron Variant |
|  | rs4482754 | chr4:86090151 | - | - | MAPK10 | Intron Variant |
|  | rs7263316 | chr20:19703392 | - | - | SLC24A3 | Intron Variant |
|  |  |  |  |  |  |  |
| Kuo et al, 2014 | rs6736615 | chr2:162717280 | - | + | KCNH7 | Intron Variant |
|  | rs7619173 | chr3:181279171 | - | - | SOX2-OT | Intron Variant |
|  | rs7651261 | chr3:181305612 | - | - | SOX2-OT | Intron Variant |
|  | rs9850867 | chr3:89083132 | - | - | None | None |
|  | rs6814785 | chr4:81347997 | - | - | None | None |
|  | rs11946541 | chr4:38749059 | - | - | LOC105374413 | Intron Variant |
|  | rs9485899 | chr6:103831989 | - | + | None | None |
|  | rs4326265 | chr6:103895261 | - | + | None | None |
|  | rs9569968 | chr13:58793923 | - | + | None | None |
|  | rs216515 | chr14:60220591 | - | - | None | None |
|  | rs2220984 | chr16:76903031 | - | - | None | None |
|  | rs7237766 | chr18:71378886 | - | + | None | None |
|  | rs7281895 | chr21:21554170 | - | + | None | None |
|  | rs1222938 | chr18:52414796 | - | - | DCC | Intron Variant |
|  | rs6736615 | chr2:162717280 | - | + | KCNH7 | Intron Variant |
|  | rs1435013 | chr2:162669601 | - | - | KCNH7 | Intron Variant |
|  | rs11001178 | chr10:74842047 | - | - | KAT6B | Intron Variant |
|  | rs16931793 | chr10:74873906 | - | - | KAT6B | Intron Variant |
|  | rs7909358 | chr10:74928324 | - | - | KAT6B | Intron Variant |
|  | rs7917746 | chr10:74905845 | - | - | KAT6B | Intron Variant |
|  | rs2217887 | chr14:79417482 | - | + | NRXN3 | Intron Variant |
|  | rs13322211 | chr3:77176746 | - | + | ROBO2 | Intron Variant |
|  | rs10260011 | chr7:85080040 | - | + | SEMA3D | Intron Variant |
|  |  |  |  |  |  |  |
| Mühleisen et al, 2014 | rs10994415 | chr10:60562276 | C | + | ANK3 | Intron Variant |
|  | rs10994397 | chr10:60519366 | T | + | ANK3 | Intron Variant |
|  | rs1938540 | chr10:60535056 | T | + | ANK3 | Intron Variant |
|  | rs12290811 | chr11:79372576 | A | + | TENM4 | Intron Variant |
|  | rs12576775 | chr11:79366149 | G | + | TENM4 | Intron Variant |
|  | rs7932890 | chr11:79357349 | G | + | TENM4 | Intron Variant |
|  | rs17826816 | chr5:7519185 | G | + | ADCY2 | Intron Variant |
|  | rs12202969 | chr6:98128347 | A | + | None | None |
|  | rs10821745 | chr10:60376448 | G | + | ANK3 | Intron Variant |
|  | rs17138171 | chr11:79351885 | C | + | TENM4 | Intron Variant |
|  | rs10821736 | chr10:60345295 | T | + | ANK3 | Intron Variant |
|  | rs6550435 | chr3:36822998 | G | + | LINC02033 | 2KB Upstream Variant |
|  | rs10994430 | chr10:60612195 | T | + | ANK3, LOC124902430 | Intron Variant, Non Coding Transcript Variant |
|  | rs10994336 | chr10:60420054 | T | + | ANK3 | Intron Variant |
|  | rs11237799 | chr11:79356427 | C | + | TENM4 | Intron Variant |
|  | rs10994308 | chr10:60339194 | A | + | ANK3 | Intron Variant |
|  | rs9834970 | chr3:36814539 | C | + | None | None |
|  | rs10509129 | chr10:60311283 | T | + | ANK3 | Intron Variant |
|  |  |  |  |  |  |  |
| Xu et al, 2014 | rs2813164 | chr1:198452278 | C | + | LOC105371677 | 500B Downstream Variant |
|  | rs11708571 | chr3:21549885 | G | - | ZNF385D, ZNF385D-AS1 | Intron Variant, Intron Variant |
|  | rs4689410 | chr4:6342477 | A | - | PPP2R2C | Intron Variant |
|  | rs215006 | chr6:152434493 | A | + | SYNE1 | Intron Variant |
|  | rs214972 | chr6:152454678 | T | + | SYNE1 | Intron Variant |
|  | rs2623971 | chr6:152509932 | A | + | SYNE1 | Intron Variant |
|  | rs2623966 | chr6:152532539 | C | + | SYNE1 | Intron Variant |
|  | rs2141150 | chr6:152547497 | C | + | SYNE1 | Intron Variant |
|  | rs2695261 | chr6:152548591 | C | + | SYNE1 | Intron Variant |
|  | rs7864144 | chr9:98643756 | G | - | GABBR2 | Intron Variant |
|  | rs12773173 | chr10:29722375 | T | - | SVIL | Intron Variant |
|  | rs1483651 | chr19:58178467 | C | + | None | None |
|  | rs4444432 | chr19:58204171 | G | + | ZNF274 | Intron Variant |
|  | rs7256349 | chr19:58206902 | A | + | ZNF274 | Missense Variant |
|  |  |  |  |  |  |  |
| Chen et al, 2013 | rs4650608 | chr1:78772330 | T | + | None | None |
|  | rs2271893 | chr2:96739703 | G | - | LMAN2L | Intron Variant |
|  | rs6746896 | chr2:96745212 | A | + | None | None |
|  | rs2875907 | chr3:85469430 | A | + | CADM2 | Intron Variant |
|  | rs17023019 | chr3:85565122 | T | + | CADM2 | Intron Variant |
|  | rs2251219 | chr3:52550771 | G | + | PBRM1, LOC124909379 | Synonymous Variant, Intron Variant |
|  | rs7618915 | chr3:52245578 | C | - | PPM1M | 2KB Upstream Variant |
|  | rs9834970 | chr3:36814539 | C | - | None | None |
|  | rs4948418 | chr10:60425736 | T | + | ANK3 | Intron Variant |
|  | rs10848642 | chr12:2222406 | G | - | CACNA1C-AS4, CACNA1C | Non Coding Transcript Variant, Intron Variant |
|  | rs6079468 | chr20:14517750 | C | - | MACROD2 | Intron Variant |
|  | rs17138171 | chr11:79351885 | A | - | TENM4 | Intron Variant |
|  | rs420259 | chr16:23622705 | A | + | PALB2 | Intron Variant |
|  | rs1012053 | chr13:42079301 | A | + | DGKH | Intron Variant |
|  | rs1006737 | chr12:2236129 | A | + | CACNA1C | Intron Variant |
|  | rs1064395 | chr19:19250926 | A | + | NCAN | 3 Prime UTR Variant |
|  | rs1344706 | chr2:184913701 | A | + | ZNF804A | Intron Variant |
|  |  |  |  |  |  |  |
| Green et al, 2013 | rs9834970 | chr3:36814539 | T | - | None | None |
|  | rs3755806 | chr3:52609669 | C | - | PBRM1 | Synonymous Variant |
|  | rs1006737 | chr12:2236129 | A | + | CACNA1C | Intron Variant |
|  | rs1274726 | chr12:49107615 | C | + | LMBR1L | Intron Variant |
|  | rs2273684 | chr20:34941963 | G | + | GSS | Intron Variant |
|  | rs3818253 | chr20:35009073 | A | + | TRPC4AP | Intron Variant |
|  | rs263906 | chr1:101512778 | C | + | None | None |
|  | rs875326 | chr1:175320263 | C | - | TNR, LOC105371623 | 3 Prime UTR Variant, Intron Variant |
|  | rs6746896 | chr2:96745212 | A | + | None | None |
|  | rs7578035 | chr2:98766429 | G | + | LINC02611 | Intron Variant |
|  | rs4668059 | chr2:168309772 | C | + | None | None |
|  | rs4332037 | chr7:1911173 | C | + | MAD1L1 | Intron Variant |
|  | rs9804190 | chr10:60080073 | C | - | ANK3 | Intron Variant |
|  | rs10994397 | chr10:60519366 | C | + | ANK3 | Intron Variant |
|  | rs12576775 | chr11:79366149 | A | - | TENM4 | Intron Variant |
|  | rs7296288 | chr12:49086185 | A | - | DHH | 500B Downstream Variant |
|  | rs16966413 | chr15:38187698 | A | - | None | None |
|  | rs12912251 | chr15:38694167 | G | + | None | None |
|  | rs2287921 | chr19:48725015 | C | + | RASIP1 | Intron Variant |
|  | rs3845817 | chr2:65531391 | C | - | None | None |
|  | rs6550435 | chr3:36822998 | G | - | LINC02033 | 2KB Upstream Variant |
|  | rs736408 | chr3:52801338 | C | + | ITIH3 | Intron Variant |
|  | rs9371601 | chr6:152469438 | G | - | SYNE1 | Intron Variant |
|  | rs3968 | chr9:4941997 | C | + | None | None |
|  | rs4765913 | chr12:2310730 | A | + | CACNA1C | Intron Variant |
|  | rs11085829 | chr19:13063498 | A | + | NFIX | Intron Variant |
|  | rs6102947 | chr20:42612212 | C | + | PTPRT | Intron Variant |
|  |  |  |  |  |  |  |
| Cichon et al, 2011 | rs1064395 | chr19:19250926 | A | + | NCAN | 3 Prime UTR Variant |
|  | rs11764590 | chr7:1993168 | T | + | MAD1L1 | Intron Variant |
|  | rs10278591 | chr7:1881726 | T | + | MAD1L1 | Intron Variant |
|  | rs6547829 | chr2:28101002 | T | + | BABAM2 | Intron Variant |
|  | rs985409 | chr7:104530976 | G | + | LHFPL3 | Intron Variant |
|  | rs2209263 | chr9:80911728 | A | - | None | None |
|  | rs779279 | chr3:191826138 | A | - | None | None |
|  | rs9322993 | chr14:39136338 | T | + | GEMIN2 | Intron Variant |
|  | rs422159 | chr17:3545428 | A | + | TRPV3 | Intron Variant |
|  | rs11577112 | chr1:18131878 | G | + | IGSF21 | Intron Variant |
|  | rs3996329 | chr7:1979235 | T | + | MAD1L1, LOC124901573 | Intron Variant, 2KB Upstream Variant |
|  | rs6488297 | chr12:10513426 | A | + | EIF2S3B, LOC105369657 | Intron Variant, Intron Variant |
|  | rs2654205 | chr15:82120057 | C | + | None | None |
|  | rs508208 | chr1:235564516 | G | - | GNG4 | Intron Variant |
|  | rs2774339 | chr1:235586278 | T | - | GNG4 | Intron Variant |
|  | rs4844367 | chrX:69559027 | A | - | None | None |
|  | rs6821225 | chr4:146081566 | C | + | None | None |
|  | rs281413 | chr19:10336058 | A | - | ICAM3 | Intron Variant |
|  | rs7297212 | chr12:5454343 | A | + | NTF3 | Intron Variant |
|  | rs930906 | chrX:19217199 | A | + | None | None |
|  | rs7023951 | chr9:114018712 | T | + | ZNF618 | Intron Variant |
|  | rs4743473 | chr9:101590292 | A | + | GRIN3A | Intron Variant |
|  | rs10500683 | chr11:7167772 | C | - | None | None |
|  | rs6506625 | chr18:8937037 | A | - | None | None |
|  | rs1435442 | chr4:183896049 | C | + | STOX2 | Intron Variant |
|  | rs502224 | chr11:64039521 | T | + | MACROD1 | Intron Variant, Intron Variant |
|  | rs16954276 | chr15:70203617 | T | - | None | None |
|  | rs1889339 | chr9:93291646 | C | + | WNK2 | Intron Variant |
|  | rs9944047 | chr14:96760486 | C | - | LINC02299 | Intron Variant |
|  | rs6943047 | chr7:55349271 | T | + | None | None |
|  | rs2398820 | chr9:93234291 | G | + | WNK2 | Intron Variant |
|  | rs11242396 | chr5:137665276 | G | + | KLHL3 | Intron Variant |
|  | rs1317266 | chr4:159506641 | G | + | None | None |
|  | rs1931364 | chr9:93285170 | A | + | WNK2 | Intron Variant |
|  | rs7229918 | chr18:73270941 | G | + | None | None |
|  | rs10426528 | chr19:43795146 | T | + | None | None |
|  | rs10231989 | chr7:93361809 | C | + | None | None |
|  | rs6715508 | chr2:204729314 | T | + | PARD3B | Intron Variant |
|  | rs1725262 | chr1:7502428 | C | + | CAMTA1, LOC105376691 | Intron Variant, 2KB Upstream Variant |
|  | rs4461243 | chr2:37534576 | A | + | LOC105374464 | Intron Variant |
|  | rs17530034 | chr2:125378695 | A | - | None | None |
|  | rs7268 | chr5:140332965 | T | + | HBEGF | 3 Prime UTR Variant |
|  | rs12123953 | chr1:78049463 | T | + | GIPC2 | Intron Variant |
|  | rs843319 | chr6:20135594 | G | + | MBOAT1 | Intron Variant |
|  | rs6767011 | chr3:36462789 | A | + | STAC | Intron Variant |
|  | rs4849303 | chr2:110970905 | T | - | ACOXL | Intron Variant |
|  | rs1203860 | chr4:2503364 | A | + | RNF4 | Intron Variant |
|  | rs705648 | chr2:216069069 | C | + | PECR | Intron Variant |
|  |  |  |  |  |  |  |
| Lee et al, 2011 | rs2709736 | chr7:20822683 | G | + | None | None |
|  | rs2709722 | chr7:20828189 | C | + | RPL23P8 | 500B Downstream Variant |
|  | rs8040009 | chr15:92501109 | C | + | C15orf32 | Non Coding Transcript Variant |
|  | rs7174854 | chr15:92500746 | T | + | C15orf32 | Non Coding Transcript Variant |
|  | rs2073831 | chr13:76924565 | T | + | None | None |
|  | rs1323038 | chr13:76923041 | T | + | None | None |
|  | rs11013860 | chr10:18365098 | A | + | CACNB2 | Intron Variant |
|  | rs576026 | chr9:18733464 | G | + | ADAMTSL1 | Intron Variant |
|  |  |  |  |  |  |  |
| Psychiatric GWAS Consortium Bipolar Disorder Working Group, 2011 | rs4765913 | chr12:2310730 | A | + | CACNA1C | Intron Variant |
|  | rs10896135 | chr11:66783531 | C | - | C11orf80 | Intron Variant |
|  | rs2070615 | chr12:48824388 | A | - | CACNB3 | Intron Variant |
|  | rs12576775 | chr11:79366149 | A | - | TENM4 | Intron Variant |
|  | rs2175420 | chr11:79412839 | C | - | TENM4 | Intron Variant |
|  | rs3845817 | chr2:65531391 | C | - | None | None |
|  | rs2176528 | chr2:194007459 | C | + | None | None |
|  | rs4660531 | chr1:41374150 | G | - | FOXO6 | Intron Variant |
|  | rs7578035 | chr2:98766429 | G | + | LINC02611 | Intron Variant |
|  | rs2287921 | chr19:48725015 | C | + | RASIP1 | Intron Variant |
|  | rs11168751 | chr12:48825355 | C | - | CACNB3 | Intron Variant |
|  | rs7296288 | chr12:49086185 | A | - | DHH | 500B Downstream Variant |
|  | rs7827290 | chr8:141290216 | G | + | SLC45A4 | Intron Variant |
|  | rs12730292 | chr1:78789077 | C | + | None | None |
|  | rs12912251 | chr15:38694167 | G | + | None | None |
|  | rs4332037 | chr7:1911173 | C | - | MAD1L1 | Intron Variant |
|  | rs6550435 | chr3:36822998 | G | + | LINC02033 | 2KB Upstream Variant |
|  | rs17395886 | chr4:161358233 | A | - | None | None |
|  | rs6746896 | chr2:96745212 | A | + | None | None |
|  | rs736408 | chr3:52801338 | C | + | ITIH3 | Intron Variant |
|  | rs11162405 | chr1:78003976 | A | - | DNAJB4 | Intron Variant |
|  | rs9804190 | chr10:60080073 | C | + | ANK3 | Intron Variant |
|  | rs9371601 | chr6:152469438 | G | - | SYNE1 | Intron Variant |
|  | rs3774609 | chr3:53798876 | G | - | CACNA1D | Intron Variant |
|  | rs10994397 | chr10:60519366 | C | - | ANK3 | Intron Variant |
|  | rs4668059 | chr2:168309772 | C | + | None | None |
|  | rs16966413 | chr15:38187698 | A | - | None | None |
|  | rs6102917 | chr20:42590779 | C | + | PTPRT | Intron Variant |
|  | rs11085829 | chr19:13063498 | A | - | NFIX | Intron Variant |
|  | rs875326 | chr1:175320263 | C | + | TNR, LOC105371623 | 3 Prime UTR Variant, Intron Variant |
|  | rs13245097 | chr7:2301420 | C | + | SNX8 | Intron Variant |
|  | rs780148 | chr10:79175326 | C | + | ZMIZ1 | Intron Variant |
|  | rs2281587 | chr10:103617592 | C | + | SH3PXD2A | Intron Variant |
|  | rs10776799 | chr1:115330426 | G | + | NGF, NGF-AS1 | Intron Variant, Intron Variant |
|  | rs263906 | chr1:101512778 | C | + | None | None |
|  | rs10028075 | chr4:86046677 | C | - | MAPK10 | Intron Variant |
|  | rs3968 | chr9:4941997 | C | + | None | None |
|  | rs8006348 | chr14:51058755 | A | - | TRIM9 | Intron Variant |
|  |  |  |  |  |  |  |
| Yosifova et al, 2011 | rs8099939 | chr19:42016956 | T | + | GRIK5 | Intron Variant |
|  | rs6122972 | chr20:50758705 | G | + | None | None |
|  | rs2289700 | chr15:78932341 | A | + | CTSH | Intron Variant |
|  | rs11129950 | chr3:42273095 | G | + | None | None |
|  | rs16970287 | chr15:78935915 | G | + | CTSH | Intron Variant |
|  | rs8109263 | chr19:20614838 | T | + | LOC105372318 | Intron Variant |
|  | rs10507408 | chr13:33514446 | G | + | STARD13 | Intron Variant |
|  | rs3123209 | chr10:131390928 | A | + | None | None |
|  | rs10491291 | chr5:133580668 | T | + | FSTL4, LOC124901066 | Intron Variant, Non Coding Transcript Variant |
|  | rs10875032 | chr1:96732775 | T | + | PTBP2 | Intron Variant |
|  |  |  |  |  |  |  |
| Djurovic et al, 2010 | rs6679053 | chr1:242122402 | C | + | PLD5 | Intron Variant |
|  | rs12052834 | chr2:116330424 | A | - | LOC105373576 | Intron Variant |
|  | rs4257412 | chr2:116367087 | T | - | LOC105373576 | Intron Variant |
|  | rs1529289 | chr2:124158066 | T | - | CNTNAP5 | Intron Variant |
|  | rs2420559 | chr2:124152424 | A | - | CNTNAP5 | Intron Variant |
|  | rs2699365 | chr2:124158584 | A | - | CNTNAP5 | Intron Variant |
|  | rs13216050 | chr6:46190316 | G | + | None | None |
|  | rs10901040 | chr7:154638563 | T | - | DPP6 | Intron Variant |
|  | rs4960568 | chr7:154639123 | T | - | DPP6 | Intron Variant |
|  | rs1510122 | chr8:77653293 | G | + | None | None |
|  | rs1567426 | chr8:77634401 | T | + | None | None |
|  | rs448245 | chr8:77600338 | G | + | None | None |
|  | rs264826 | chr8:77458426 | A | + | LOC102724874 | Intron Variant |
|  | rs7844514 | chr8:97117952 | T | + | CPQ, LOC101927066 | Intron Variant |
|  | rs2578121 | chr10:78457451 | C | + | LOC107984245 | Intron Variant |
|  | rs1435961 | chr10:78461228 | A | + | LOC107984245 | Non Coding Transcript Variant |
|  | rs7100273 | chr10:53650241 | T | - | None | None |
|  | rs7081438 | chr10:53697475 | T | - | None | None |
|  | rs7119726 | chr11:70612743 | T | + | SHANK2 | Intron Variant |
|  | rs7310876 | chr12:13406433 | G | + | None | None |
|  | rs1750565 | chr13:50164164 | G | - | DLEU1 | Intron Variant |
|  | rs1798968 | chr13:50163733 | T | - | DLEU1 | Intron Variant |
|  | rs809846 | chr13:50174395 | A | - | DLEU1 | Intron Variant |
|  | rs1750567 | chr13:50162887 | G | - | DLEU1 | Intron Variant |
|  | rs11617400 | chr13:51003439 | C | + | GUCY1B2 | Intron Variant |
|  | rs9527256 | chr13:54682204 | C | - | None | None |
|  | rs8042197 | chr15:48656094 | C | - | None | None |
|  | rs1848053 | chr15:48655765 | G | - | None | None |
|  | rs931781 | chr15:48662707 | A | - | None | None |
|  | rs7183870 | chr15:92057169 | A | - | SLCO3A1 | Intron Variant |
|  | rs7184694 | chr16:79657421 | T | - | None | None |
|  | rs11652429 | chr17:34180578 | C | + | LINC01989 | Intron Variant |
|  | rs11080256 | chr17:34181763 | A | + | LINC01989 | Intron Variant |
|  | rs11658512 | chr17:34180592 | A | + | LINC01989 | Intron Variant |
|  | rs1292735 | chr21:14730332 | G | - | None | None |
|  |  |  |  |  |  |  |
| Scott et al, 2009 | rs472913 | chr1:60629886 | C | + | None | None |
|  | rs1042779 | chr3:52786995 | A | + | ITIH1 | Missense Variant |
|  | rs17418283 | chr5:94818883 | C | + | MCTP1 | Intron Variant |
|  |  |  |  |  |  |  |
| Ferreira et al, 2008 | rs10994336 | chr10:60420054 | T | + | ANK3 | Intron Variant |
|  | rs1938526 | chr10:60540625 | G | + | ANK3 | Intron Variant |
|  | rs1006737 | chr12:2236129 | A | + | CACNA1C | Intron Variant |
|  | rs1024582 | chr12:2293080 | A | + | CACNA1C | Intron Variant |
|  | rs12899449 | chr15:38703290 | G | - | None | None |
|  | rs2172835 | chr15:38697970 | T | - | LINC02694 | Intron Variant |

**Supplementary Table 2: The List of significant single nucleotide polymorphism and genes of dementia by the genome-wide association study**

| Author | SNPs | Position | Minor Alleles | Allele Directions | Gene | Consequence |
| --- | --- | --- | --- | --- | --- | --- |
| Dalmasso et al, 2024 | rs2820864 | chr1:163485057 | C | - | None | None |
|  | rs36039096 | chr2:35789890 | A | - | None | None |
|  | rs35392935 | chr2:40071018 | T | + | SLC8A1-AS1 | Intron Variant |
|  | rs7595509 | chr2:67888895 | A | - | None | None |
|  | rs12465126 | chr2:235676849 | A | + | AGAP1 | Intron Variant |
|  | rs553467 | chr5:6573819 | A | + | None | None |
|  | rs29745 | chr5:31656661 | A | - | PDZD2 | Intron Variant |
|  | rs7016182 | chr8:77958623 | C | + | None | None |
|  | rs74457370 | chr9:92567110 | A | - | CENPP | Intron Variant |
|  | rs2805792 | chr9:97591519 | T | - | TMOD1 | Intron Variant |
|  | rs57464688 | chr9:134858932 | A | + | LOC101448202 | Intron Variant |
|  | rs9566005 | chr13:85053369 | C | - | None | None |
|  | rs949937 | chr14:20490566 | A | - | None | None |
|  | rs8103283 | chr19:1103523 | A | - | GPX4 | 2KB Upstream Variant |
|  | rs34532322 | chr21:34364698 | A | + | KCNE2 | Intron Variant |
|  | rs10169262 | chr2:9409624 | T | - | ITGB1BP1 | Intron Variant |
|  | rs376291994 | chr4:59984152 | A | - | None | None |
|  | rs1531681 | chr6:28259100 | A | - | ZKSCAN4, NKAPL | Intron Variant, 2KB Upstream Variant |
|  | rs115038899 | chr8:125446584 | T | + | None | None |
|  | rs11849532 | chr14:106669877 | A | - | None | None |
|  | rs61392417 | chr18:32075509 | T | - | RNF125 | Intron Variant |
|  |  |  |  |  |  |  |
| Sherva et al, 2023 | rs116329346 | chr2:38516744 | A | + | LINC02613 | 2KB Upstream Variant |
|  | rs58443395 | chr2:53254996 | A | - | None | None |
|  | rs10197243 | chr2:54825356 | T | + | EML6 | Intron Variant |
|  | rs567572378 | chr3:19591973 | A | + | None | None |
|  | rs11919682 | chr3:78720952 | C | - | ROBO1 | Intron Variant |
|  | rs192764155 | chr4:58928804 | A | - | LINC02619 | Intron Variant |
|  | rs148433063 | chr4:59020931 | T | - | LINC02429 | Intron Variant |
|  | rs28377689 | chr4:80046517 | T | - | ANTXR2 | Intron Variant |
|  | rs74852218 | chr5:25701113 | A | + | None | None |
|  | rs114681435 | chr5:38361439 | T | - | EGFLAM | Intron Variant |
|  | rs2234253 | chr6:41161367 | T | + | TREM2 | Missense Variant |
|  | rs73427293 | chr6:41168873 | A | - | None | None |
|  | rs16894668 | chr6:41421172 | T | + | None | None |
|  | rs7738720 | chr6:47427663 | T | - | None | None |
|  | rs4607615 | chr8:10422116 | C | - | MSRA | Intron Variant |
|  | rs73581622 | chr8:31400910 | A | + | LOC101929492 | Non Coding Transcript Variant |
|  | rs112395375 | chr8:104686541 | A | + | None | None |
|  | rs76427927 | chr8:115111058 | A | - | None | None |
|  | rs509334 | chr11:121407209 | A | + | None | None |
|  | rs145008711 | chr15:68433764 | A | - | ITGA11 | 2KB Upstream Variant |
|  | rs116620371 | chr18:36600029 | A | - | FHOD3 | Intron Variant |
|  | rs73505251 | chr19:1068096 | A | + | ARHGAP45 | Intron Variant |
|  | rs429358 | chr19:44908684 | T | - | APOE | Missense Variant |
|  |  |  |  |  |  |  |
| Bellenguez et al, 2022 | rs679515 | chr1:207577223 | T | + | CR1 | Intron Variant |
|  | rs6733839 | chr2:127135234 | T | + | LOC105373605 | Intron Variant |
|  | rs10933431 | chr2:233117202 | G | - | INPP5D | Intron Variant |
|  | rs6846529 | chr4:11023507 | C | + | None | None |
|  | rs6605556 | chr6:32615322 | G | - | None | None |
|  | rs10947943 | chr6:41036354 | A | - | UNC5CL | Intron Variant |
|  | rs143332484 | chr6:41161469 | T | + | TREM2 | Missense Variant |
|  | rs75932628 | chr6:41161514 | T | + | TREM2 | Missense Variant |
|  | rs60755019 | chr6:41181270 | G | + | LOC107986595 | Intron Variant |
|  | rs7767350 | chr6:47517390 | T | + | CD2AP | Intron Variant |
|  | rs6966331 | chr7:37844191 | T | - | None | None |
|  | rs7384878 | chr7:100334426 | C | - | STAG3L5P-PVRIG2P-PILRB, PMS2P1, STAG3L5P | 2KB Upstream Variant, Intron Variant, 2KB Upstream Variant |
|  | rs11771145 | chr7:143413669 | A | - | EPHA1-AS1 | Intron Variant |
|  | rs73223431 | chr8:27362470 | T | + | PTK2B | Intron Variant |
|  | rs11787077 | chr8:27607795 | T | - | CLU | Intron Variant |
|  | rs7912495 | chr10:11676714 | G | + | LOC105376412, LOC105376413 | Non Coding Transcript Variant, Intron Variant |
|  | rs10437655 | chr11:47370397 | A | + | SPI1 | Intron Variant |
|  | rs1582763 | chr11:60254475 | A | - | None | None |
|  | rs3851179 | chr11:86157598 | T | - | None | None |
|  | rs74685827 | chr11:121482368 | G | + | SORL1, LOC105369535 | Intron Variant, 2KB Upstream Variant |
|  | rs11218343 | chr11:121564878 | C | - | SORL1 | Intron Variant |
|  | rs17125924 | chr14:52924962 | G | + | FERMT2, LOC105370500 | Intron Variant, Intron Variant |
|  | rs7401792 | chr14:92464917 | G | + | SLC24A4 | Intron Variant |
|  | rs12590654 | chr14:92472511 | A | - | SLC24A4 | Intron Variant |
|  | rs8025980 | chr15:50701814 | G | - | SPPL2A | 500B Downstream Variant |
|  | rs602602 | chr15:58764824 | A | - | None | None |
|  | rs117618017 | chr15:63277703 | T | + | APH1B | Missense Variant |
|  | rs889555 | chr16:31111250 | T | - | BCKDK | Intron Variant |
|  | rs4985556 | chr16:70660097 | A | + | IL34 | Stop Gained |
|  | rs12446759 | chr16:81739398 | G | - | None | None |
|  | rs72824905 | chr16:81908423 | G | - | PLCG2 | Missense Variant |
|  | rs7225151 | chr17:5233752 | A | + | SCIMP, ZNF594-DT | Intron Variant, Intron Variant |
|  | rs199515 | chr17:46779275 | G | - | LRRC37A2, WNT3 | Intron Variant, Intron Variant |
|  | rs616338 | chr17:49219935 | T | + | ABI3 | Missense Variant |
|  | rs2526377 | chr17:58332680 | G | - | TSPOAP1-AS1, MIR142 | Intron Variant, 2KB Upstream Variant |
|  | rs4277405 | chr17:63471557 | C | - | None | None |
|  | rs12151021 | chr19:1050875 | A | + | ABCA7 | Intron Variant |
|  | rs6014724 | chr20:56423488 | G | - | CASS4 | Intron Variant |
|  | rs2830489 | chr21:26775872 | T | - | None | None |
|  | rs141749679 | chr1:109345810 | C | + | SORT1 | Missense Variant |
|  | rs72777026 | chr2:9558882 | G | + | None | None |
|  | rs17020490 | chr2:37304796 | C | + | PRKD3 | Intron Variant |
|  | rs143080277 | chr2:105749599 | C | + | NCK2 | Intron Variant |
|  | rs139643391 | chr2:202878717 | T | - | WDR12 | 3 Prime UTR Variant |
|  | rs16824536 | chr3:155069722 | A | - | MME | Intron Variant |
|  | rs61762319 | chr3:155084189 | G | + | MME | Missense Variant |
|  | rs3822030 | chr4:993555 | G | - | IDUA, SLC26A1 | Intron Variant, 2KB Upstream Variant |
|  | rs2245466 | chr4:40197226 | G | + | RHOH | Intron Variant |
|  | rs112403360 | chr5:14724304 | A | + | ANKH, LOC124900944 | Intron Variant, Non Coding Transcript Variant |
|  | rs62374257 | chr5:86927378 | C | + | None | None |
|  | rs871269 | chr5:151052827 | T | - | TNIP1 | Intron Variant |
|  | rs113706587 | chr5:180201150 | A | + | RASGEF1C | Intron Variant |
|  | rs785129 | chr6:114291731 | T | + | HS3ST5, HDAC2-AS2 | Intron Variant, Intron Variant |
|  | rs6943429 | chr7:7817263 | T | + | UMAD1 | Intron Variant |
|  | rs10952097 | chr7:8204382 | T | + | ICA1 | Intron Variant |
|  | rs13237518 | chr7:12229967 | A | - | TMEM106B | Intron Variant |
|  | rs1160871 | chr7:28129127-28129134 | G | - | JAZF1 | Intron Variant |
|  | rs76928645 | chr7:54873635 | T | - | None | None |
|  | rs1065712 | chr8:11844613 | C | + | CTSB | 3 Prime UTR Variant |
|  | rs34173062 | chr8:144103704 | A | + | SHARPIN, MAF1 | Missense Variant, 2KB Upstream Variant |
|  | rs1800978 | chr9:104903697 | G | + | ABCA1 | 5 Prime UTR Variant |
|  | rs7068231 | chr10:60025170 | T | - | None | None |
|  | rs6586028 | chr10:80494228 | C | - | TSPAN14 | Intron Variant |
|  | rs6584063 | chr10:96266650 | G | - | BLNK | Intron Variant |
|  | rs7908662 | chr10:122413396 | G | - | PLEKHA1 | Intron Variant |
|  | rs6489896 | chr12:113281983 | C | + | TPCN1 | Intron Variant |
|  | rs7157106 | chr14:105761758 | A | + | LOC105378184 | Intron Variant |
|  | rs10131280 | chr14:106665591 | A | - | LOC102724977 | Intron Variant |
|  | rs3848143 | chr15:64131307 | G | + | SNX1 | Intron Variant |
|  | rs12592898 | chr15:78936857 | A | - | CTSH | Intron Variant |
|  | rs1140239 | chr16:30010081 | T | - | DOC2A | Missense Variant |
|  | rs450674 | chr16:79574511 | C | - | MAF | Intron Variant |
|  | rs16941239 | chr16:86420604 | A | + | None | None |
|  | rs56407236 | chr16:90103687 | A | + | FAM157C, LOC105376786 | Intron Variant, 2KB Upstream Variant |
|  | rs35048651 | chr17:1728047-1728058 | T | + | WDR81 | Inframe Deletion |
|  | rs2242595 | chr17:18156140 | A | - | MYO15A, LOC105371567 | Intron Variant, 2KB Upstream Variant |
|  | rs5848 | chr17:44352876 | T | + | GRN, FAM171A2 | 3 Prime UTR Variant, 500B Downstream Variant |
|  | rs149080927 | chr19:1854255-1854259 | G | + | KLF16 | 3 Prime UTR Variant |
|  | rs9304690 | chr19:49950060 | T | + | SIGLEC11, LOC124904802 | Synonymous Variant, 2KB Upstream Variant |
|  | rs587709 | chr19:54267597 | C | + | LAIR1 | Intron Variant |
|  | rs1358782 | chr20:413334 | A | - | RBCK1 | Intron Variant |
|  | rs6742 | chr20:63743088 | T | - | SLC2A4RG | 3 Prime UTR Variant |
|  | rs2154481 | chr21:26101558 | C | - | APP | Intron Variant |
|  |  |  |  |  |  |  |
| Harper et al, 2022 | rs429358 | chr19:44908684 | C | + | APOE | Missense Variant |
|  | rs4420638 | chr19:44919689 | G | + | APOC1 | 500B Downstream Variant |
|  | rs12721051 | chr19:44918903 | G | + | APOC1 | Intron Variant |
|  | rs769449 | chr19:44906745 | A | + | APOE | Intron Variant |
|  | rs6857 | chr19:44888997 | T | + | NECTIN2 | 3 Prime UTR Variant |
|  | rs7256200 | chr19:44912678 | T | + | APOC1 | 2KB Upstream Variant |
|  | rs192213585 | chr12:28765164 | C | + | LOC105369711 | Intron Variant |
|  | rs148760255 | chr12:28602488 | G | + | None | None |
|  | rs148377161 | chr12:28677164 | T | + | None | None |
|  | rs76679949 | chr5:168793635 | C | + | SLIT3 | Intron Variant |
|  | rs147211831 | chr9:27436086 | A | + | MOB3B | Intron Variant |
|  | rs117204439 | chr9:27607975 | C | + | None | None |
|  |  |  |  |  |  |  |
| Wightman et al, 2021 | rs113020870 | chr1:985377 | T | + | AGRN | Synonymous Variant |
|  | rs679515 | chr1:207577223 | T | + | CR1 | Intron Variant |
|  | rs115186657 | chr2:105618971 | C | + | None | None |
|  | rs4663105 | chr2:127133851 | C | + | LOC105373605 | Intron Variant |
|  | rs7597763 | chr2:233173931 | C | + | INPP5D | Intron Variant |
|  | rs4504245 | chr4:11013198 | G | + | None | None |
|  | rs871269 | chr5:151052827 | T | - | TNIP1 | Intron Variant |
|  | rs6891966 | chr5:157099320 | G | + | HAVCR2 | Intron Variant |
|  | rs1846190 | chr6:32616036 | A | + | None | None |
|  | rs187370608 | chr6:40974457 | G | - | LOC101929555 | Intron Variant |
|  | rs9369716 | chr6:47584444 | T | + | CD2AP | Intron Variant |
|  | rs5011436 | chr7:12229132 | C | + | TMEM106B | Intron Variant |
|  | rs7384878 | chr7:100334426 | T | + | STAG3L5P-PVRIG2P-PILRB, PMS2P1, STAG3L5P | 2KB Upstream Variant, Intron Variant, 2KB Upstream Variant |
|  | rs3935067 | chr7:143407238 | G | + | EPHA1, EPHA1-AS1 | Intron Variant, 2KB Upstream Variant |
|  | rs1532278 | chr8:27608798 | T | + | CLU | Intron Variant |
|  | rs61732533 | chr8:144053248 | G | + | OPLAH | Missense Variant |
|  | rs7912495 | chr10:11676714 | G | + | LOC105376412, LOC105376413 | Non Coding Transcript Variant, Intron Variant |
|  | rs7902657 | chr10:59978394 | T | + | None | None |
|  | rs3740688 | chr11:47358789 | T | + | SPI1 | Intron Variant |
|  | rs1582763 | chr11:60254475 | G | + | None | None |
|  | rs561655 | chr11:86089237 | G | + | None | None |
|  | rs11218343 | chr11:121564878 | T | + | SORL1 | Intron Variant |
|  | rs7146179 | chr14:52832135 | G | + | LOC105370500 | Intron Variant |
|  | rs12590654 | chr14:92472511 | G | + | SLC24A4 | Intron Variant |
|  | rs602602 | chr15:58764824 | T | + | None | None |
|  | rs117618017 | chr15:63277703 | T | + | APH1B | Missense Variant |
|  | rs7209200 | chr17:5066645 | T | + | None | None |
|  | rs708382 | chr17:44364976 | T | + | FAM171A2 | 2KB Upstream Variant |
|  | rs28394864 | chr17:49373413 | A | + | ZNF652-AS1 | Intron Variant |
|  | rs2632516 | chr17:58331728 | G | + | TSPOAP1-AS1, MIR142 | Intron Variant, 2KB Upstream Variant |
|  | rs6504163 | chr17:63468418 | T | + | None | None |
|  | rs12151021 | chr19:1050875 | A | + | ABCA7 | Intron Variant |
|  | rs429358 | chr19:44908684 | C | + | APOE | Missense Variant |
|  | rs2452170 | chr19:48710247 | G | + | MAMSTR | Intron Variant |
|  | rs1354106 | chr19:51234736 | G | + | CD33, LOC107985327 | Intron Variant, Intron Variant |
|  | rs1761461 | chr19:54313903 | C | + | LILRA5 | 2KB Upstream Variant |
|  | rs6069737 | chr20:56420643 | T | + | CASS4 | Intron Variant |
|  | rs2154482 | chr21:26148613 | T | + | APP, LOC124900466 | Intron Variant, Intron Variant |
|  |  |  |  |  |  |  |
| Jansen et al, 2019 | rs4575098 | chr1:161185602 | A | + | ADAMTS4 | 3 Prime UTR Variant |
|  | rs2093760 | chr1:207613483 | A | + | CR1 | Intron Variant |
|  | rs4663105 | chr2:127133851 | C | + | LOC105373605 | Intron Variant |
|  | rs10933431 | chr2:233117202 | G | - | INPP5D | Intron Variant |
|  | rs184384746 | chr3:57192122 | T | + | None | None |
|  | rs6448453 | chr4:11024404 | A | + | None | None |
|  | rs7657553 | chr4:11721611 | A | + | LOC107986178 | Intron Variant |
|  | rs6931277 | chr6:32615580 | T | - | None | None |
|  | rs187370608 | chr6:40974457 | A | + | LOC101929555 | Intron Variant |
|  | rs9381563 | chr6:47464901 | C | + | None | None |
|  | rs1859788 | chr7:100374211 | A | - | PILRA | Missense Variant |
|  | rs7810606 | chr7:143411065 | T | - | EPHA1-AS1 | Intron Variant |
|  | rs114360492 | chr7:146252937 | T | + | CNTNAP2 | Intron Variant |
|  | rs4236673 | chr8:27607412 | A | - | CLU | Intron Variant |
|  | rs11257238 | chr10:11675398 | C | + | LOC105376412, LOC105376413 | Intron Variant, Intron Variant |
|  | rs2081545 | chr11:60190907 | A | - | None | None |
|  | rs867611 | chr11:86065502 | G | - | PICALM, LOC124902730 | Intron Variant, 2KB Upstream Variant |
|  | rs11218343 | chr11:121564878 | C | - | SORL1 | Intron Variant |
|  | rs12590654 | chr14:92472511 | A | - | SLC24A4 | Intron Variant |
|  | rs442495 | chr15:58730416 | C | - | ADAM10 | Intron Variant |
|  | rs117618017 | chr15:63277703 | T | + | APH1B | Missense Variant |
|  | rs59735493 | chr16:31121779 | A | - | KAT8 | Intron Variant |
|  | rs113260531 | chr17:5235685 | A | + | ZNF594-DT, SCIMP | Non Coding Transcript Variant, 2KB Upstream Variant |
|  | rs28394864 | chr17:49373413 | A | + | ZNF652-AS1 | Intron Variant |
|  | rs2632516 | chr17:58331728 | C | - | TSPOAP1-AS1, MIR142 | Intron Variant, 2KB Upstream Variant |
|  | rs8093731 | chr18:31508995 | T | - | DSG2 | Intron Variant |
|  | rs76726049 | chr18:58522227 | C | + | ALPK2 | Intron Variant |
|  | rs111278892 | chr19:1039324 | G | + | ABCA7, CNN2 | 2KB Upstream Variant, 500B Downstream Variant |
|  | rs41289512 | chr19:44848259 | G | + | NECTIN2 | Intron Variant |
|  | rs76320948 | chr19:45738583 | T | + | MEIOSIN | Intron Variant |
|  | rs3865444 | chr19:51224706 | A | - | CD33, LOC107985327 | Non Coding Transcript Variant, Intron Variant |
|  | rs6014724 | chr20:56423488 | G | - | CASS4 | Intron Variant |
|  |  |  |  |  |  |  |
| Kunkle et al, 2019 | rs4844610 | chr1:207629207 | A | + | CR1 | Intron Variant |
|  | rs6733839 | chr2:127135234 | T | + | LOC105373605 | Intron Variant |
|  | rs10933431 | chr2:233117202 | G | - | INPP5D | Intron Variant |
|  | rs9271058 | chr6:32607629 | A | + | None | None |
|  | rs75932628 | chr6:41161514 | T | + | TREM2 | Missense Variant |
|  | rs9473117 | chr6:47463548 | C | + | None | None |
|  | rs12539172 | chr7:100494172 | T | - | NYAP1 | 3 Prime UTR Variant |
|  | rs10808026 | chr7:143402040 | A | - | EPHA1 | Intron Variant |
|  | rs73223431 | chr8:27362470 | T | + | PTK2B | Intron Variant |
|  | rs9331896 | chr8:27610169 | C | - | CLU, MIR6843 | Intron Variant, 500B Downstream Variant |
|  | rs3740688 | chr11:47358789 | G | - | SPI1 | Intron Variant |
|  | rs7933202 | chr11:60169453 | C | - | None | None |
|  | rs3851179 | chr11:86157598 | T | - | None | None |
|  | rs11218343 | chr11:121564878 | C | - | SORL1 | Intron Variant |
|  | rs17125924 | chr14:52924962 | G | + | FERMT2, LOC105370500 | Intron Variant, Intron Variant |
|  | rs12881735 | chr14:92466484 | C | - | SLC24A4 | Intron Variant |
|  | rs3752246 | chr19:1056493 | G | + | ABCA7 | Missense Variant |
|  | rs429358 | chr19:44908684 | C | + | APOE | Missense Variant |
|  | rs6024870 | chr20:56422512 | A | - | CASS4 | Intron Variant |
|  | rs7920721 | chr10:11678309 | G | + | LOC105376412, LOC105376413 | Intron Variant, Intron Variant |
|  | rs138190086 | chr17:63460787 | A | + | None | None |
|  | rs190982 | chr5:88927603 | G | - | MEF2C-AS1 | Intron Variant |
|  | rs4723711 | chr7:37804661 | T | - | None | None |
|  | rs4735340 | chr8:94964023 | A | - | NDUFAF6 | Intron Variant |
|  | rs7920721 | chr10:11678309 | G | + | LOC105376412,LOC105376413 | Intron Variant, Intron Variant |
|  | rs7295246 | chr12:43573874 | G | + | None | None |
|  | rs10467994 | chr15:50716490 | C | - | SPPL2A | Intron Variant |
|  | rs593742 | chr15:58753575 | G | - | None | None |
|  | rs7185636 | chr16:19796841 | C | - | IQCK | Intron Variant |
|  | rs2632516 | chr17:58331728 | C | - | TSPOAP1-AS1, MIR142 | Intron Variant, 2KB Upstream Variant |
|  | rs138190086 | chr17:63460787 | A | + | None | None |
|  | rs2830500 | chr21:26784537 | A | - | None | None |
|  | rs71618613 | chr5:29005878 | C | - | None | None |
|  | rs35868327 | chr5:53369400 | A | - | None | None |
|  | rs114812713 | chr6:41066261 | C | + | OARD1 | 3 Prime UTR Variant |
|  | rs62039712 | chr16:79321960 | A | + | MAF | Intron Variant |
|  |  |  |  |  |  |  |
| Moreno-Grau et al, 2019 | rs117834366 | chr7:147937799 | A | + | CNTNAP2 | Intron Variant |
|  | rs4704171 | chr5:75072429 | C | + | ANKRD31 | Intron Variant |
|  | rs10098778 | chr8:94979792 | C | - | NDUFAF6 | Intron Variant |
|  | rs7225151 | chr17:5233752 | A | + | SCIMP, ZNF594-DT | Intron Variant, Intron Variant |
|  |  |  |  |  |  |  |
| Witoelar et al, 2018 | rs6448807 | chr4:11674520 | C | + | LOC107986178 | Intron Variant |
|  | rs7920721 | chr10:11678309 | A | + | LOC105376412, LOC105376413 | Intron Variant, Intron Variant |
|  | rs79452530 | chr14:106699992 | C | - | None | None |
|  | rs2526378 | chr17:58326988 | A | - | TSPOAP1-AS1, TSPOAP1 | Intron Variant, Intron Variant |
|  | rs12138394 | chr1:193679698 | T | + | LOC124904475 | Intron Variant |
|  | rs7419666 | chr2:233138649 | C | + | INPP5D | Intron Variant |
|  | rs13133131 | chr4:11717374 | T | + | LOC107986178 | Intron Variant |
|  | rs13257021 | chr8:94953467 | G | + | NDUFAF6 | Intron Variant |
|  | rs7920721 | chr10:11678309 | G | + | LOC105376412, LOC105376413 | Intron Variant, Intron Variant |
|  | rs78631692 | chr14:106709371 | T | - | None | None |
|  | rs593742 | chr15:58753575 | G | - | None | None |
|  | rs2632516 | chr17:58331728 | C | - | TSPOAP1-AS1, MIR142 | Intron Variant, 2KB Upstream Variant |
|  | rs6656401 | chr1:207518704 | A | + | CR1 | Intron Variant |
|  | rs6733839 | chr2:127135234 | T | + | LOC105373605 | Intron Variant |
|  | rs9473117 | chr6:47463548 | C | + | None | None |
|  | rs2718058 | chr7:37801932 | G | - | None | None |
|  | rs1859788 | chr7:100374211 | A | - | PILRA | Missense Variant |
|  | rs11771145 | chr7:143413669 | A | - | EPHA1-AS1 | Intron Variant |
|  | rs7982 | chr8:27604964 | A | - | CLU | Missense Variant |
|  | rs3740688 | chr11:47358789 | G | - | SPI1 | Intron Variant |
|  | rs983392 | chr11:60156035 | G | - | None | None |
|  | rs10792832 | chr11:86156833 | A | - | None | None |
|  | rs11218343 | chr11:121564878 | C | - | SORL1 | Intron Variant |
|  | rs17125944 | chr14:52933911 | C | + | FERMT2 | Intron Variant |
|  | rs10498633 | chr14:92460608 | T | - | SLC24A4 | Intron Variant |
|  | rs3752246 | chr19:1056493 | G | + | ABCA7 | Missense Variant |
|  | rs7274581 | chr20:56443204 | C | - | CASS4 | Intron Variant |
|  |  |  |  |  |  |  |
| Jun et al, 2016 | rs16847609 | chr3:137931894 | A | + | None | None |
|  | rs382216 | chr5:131351444 | T | - | CDC42SE2 | Intron Variant |
|  | rs11168036 | chr5:140327854 | T | - | None | None |
|  | rs2732703 | chr17:46275856 | G | - | LRRC37A, ARL17B, LOC124904014 | Intron Variant, Intron Variant, Intron Variant |
|  | rs71380849 | chr17:74979653 | A | + | None | None |
|  | rs679515 | chr1:207577223 | T | + | CR1 | Intron Variant |
|  | rs4663105 | chr2:127133851 | C | + | LOC105373605 | Intron Variant |
|  | rs9331896 | chr8:27610169 | C | - | CLU, MIR6843 | Intron Variant, 500B Downstream Variant |
|  | rs1582763 | chr11:60254475 | A | - | None | None |
|  |  |  |  |  |  |  |
| Lambert et al, 2013 | rs6656401 | chr1:207518704 | A | + | CR1 | Intron Variant |
|  | rs6733839 | chr2:127135234 | T | + | LOC105373605 | Intron Variant |
|  | rs10948363 | chr6:47520026 | G | + | CD2AP | Intron Variant |
|  | rs11771145 | chr7:143413669 | A | - | EPHA1-AS1 | Intron Variant |
|  | rs9331896 | chr8:27610169 | C | - | CLU, MIR6843 | Intron Variant, 500B Downstream Variant |
|  | rs983392 | chr11:60156035 | G | - | None | None |
|  | rs10792832 | chr11:86156833 | A | - | None | None |
|  | rs4147929 | chr19:1063444 | A | + | ABCA7 | Intron Variant |
|  | rs3865444 | chr19:51224706 | A | - | CD33, LOC107985327 | Non Coding Transcript Variant, Intron Variant |
|  | rs9271192 | chr6:32610753 | C | + | None | None |
|  | rs28834970 | chr8:27337604 | C | + | PTK2B | Intron Variant |
|  | rs11218343 | chr11:121564878 | C | - | SORL1 | Intron Variant |
|  | rs10498633 | chr14:92460608 | T | - | SLC24A4 | Intron Variant |
|  | rs8093731 | chr18:31508995 | T | - | DSG2 | Intron Variant |
|  | rs35349669 | chr2:233159830 | T | + | INPP5D | Intron Variant |
|  | rs190982 | chr5:88927603 | G | - | MEF2C-AS1 | Intron Variant |
|  | rs2718058 | chr7:37801932 | G | - | None | None |
|  | rs1476679 | chr7:100406823 | C | - | ZCWPW1 | Intron Variant |
|  | rs10838725 | chr11:47536319 | C | + | CELF1 | Intron Variant |
|  | rs17125944 | chr14:52933911 | C | + | FERMT2 | Intron Variant |
|  | rs7274581 | chr20:56443204 | C | - | CASS4 | Intron Variant |
|  |  |  |  |  |  |  |
| Seshadri et al, 2010 | rs2075650 | chr19:44892362 | G | + | TOMM40 | Intron Variant |
|  | rs11136000 | chr8:27607002 | T | - | CLU | Intron Variant |
|  | rs3851179 | chr11:86157598 | T | - | None | None |
|  | rs744373 | chr2:127137039 | G | + | None | None |
|  | rs597668 | chr19:45205630 | C | + | BLOC1S3 | Intron Variant |
|  | rs11771145 | chr7:143413669 | A | - | EPHA1-AS1 | Intron Variant |
|  | rs2043948 | chr14:74606345 | T | + | LTBP2 | Intron Variant |
|  | rs2825544 | chr21:19368235 | C | + | None | None |
|  | rs7527934 | chr1:14031929 | G | - | KAZN | Intron Variant |
|  | rs4296166 | chr14:32483161 | A | + | AKAP6 | Intron Variant |
|  |  |  |  |  |  |  |
| Mukherjee et al, 2020 | rs1977412 | chr1:230716523 | T | - | AGT | Intron Variant |
|  | rs9937469 | chr16:9158799 | T | + | None | None |
|  | rs2795228 | chr1:83475611 | A | - | None | None |
|  | rs484947 | chr1:229029741 | A | - | None | None |
|  | rs16839220 | chr2:155547356 | C | - | LOC107985953 | Intron Variant |
|  | rs2289506 | chr3:100346058 | T | + | NIT2 | Intron Variant |
|  | rs9369477 | chr6:44340892 | T | - | POLR1C, SPATS1 | Intron Variant, 2KB Upstream Variant |
|  | rs9372110 | chr6:105869321 | A | + | LOC105377923 | Intron Variant |
|  | rs2046197 | chr8:3762230 | C | + | CSMD1 | Intron Variant |
|  | rs8021600 | chr14:43701423 | C | - | None | None |
|  | rs8091629 | chr18:45640366 | A | - | SLC14A2, LOC105372093 | Intron Variant, Intron Variant |
|  | rs13374908 | chr1:179780639 | A | + | FAM163A | Intron Variant |
|  | rs28715896 | chr2:211695376 | C | - | ERBB4 | Intron Variant |
|  | rs75337321 | chr3:54550560 | T | + | CACNA2D3 | Intron Variant |
|  | rs10222981 | chr4:6759326 | T | + | None | None |
|  | rs61835453 | chr10:2717604 | T | - | None | None |
|  | rs365521 | chr17:62944934 | A | - | None | None |
|  | rs698842 | chr2:50662958 | A | + | NRXN1 | Intron Variant |
|  | rs10175975 | chr2:59202672 | T | + | None | None |
|  | rs78872508 | chr7:18976853 | T | - | HDAC9 | Intron Variant |
|  | rs4348488 | chr8:22166649 | C | + | BMP1 | Intron Variant |
|  | rs17089546 | chr8:23714294 | A | + | LOC101929258, LOC124901910, LOC107986930 | Non Coding Transcript Variant, Non Coding Transcript Variant, Intron Variant |
|  | rs74717330 | chr8:75384073 | A | + | None | None |
|  | rs191325450 | chr9:80763069 | A | - | None | None |
|  | rs4543939 | chr11:92934492 | A | + | None | None |
|  | rs8059356 | chr16:86323639 | A | + | None | None |
|  | rs4972634 | chr2:174042477 | T | + | None | None |
|  | rs11708767 | chr3:151401938 | A | + | MED12L | Intron Variant |
|  | rs4533991 | chr6:51233850 | T | + | None | None |
|  | rs78358979 | chr6:86795185 | A | + | None | None |
|  | rs6978679 | chr7:106078297 | A | + | None | None |
|  | rs72839770 | chr17:7228873 | T | + | DVL2 | Intron Variant |
|  | rs7264688 | chr20:17974063 | T | + | MGME1 | Intron Variant |
|  |  |  |  |  |  |  |
| Beecham et al, 2009 | rs1415985 | chr1:49465077 | - | - | AGBL4, AGBL4-IT1 | Intron Variant, Intron Variant |
|  | rs11205641 | chr1:49719403 | - | - | AGBL4 | Intron Variant |
|  | rs4926831 | chr1:49824429 | - | - | AGBL4 | Intron Variant |
|  | rs9659092 | chr1:49977917 | - | - | AGBL4 | Intron Variant |
|  | rs11583200 | chr1:50094148 | - | - | ELAVL4 | Intron Variant |
|  | rs11683103 | chr2:34687783 | - | - | LINC01320 | Intron Variant |
|  | rs2119067 | chr2:165270773 | - | - | SCN2A | Intron Variant |
|  | rs10184275 | chr2:165271418 | - | - | SCN2A | Intron Variant |
|  | rs2681411 | chr3:122066784 | - | - | CD86 | Intron Variant |
|  | rs12639920 | chr4:42410670 | - | - | ATP8A1 | 3 Prime UTR Variant |
|  | rs3807031 | chr6:30066107 | - | - | PPP1R11 | Intron Variant |
|  | rs929156 | chr6:30171922 | - | - | TRIM15 | Missense Variant |
|  | rs11754661 | chr6:150885942 | - | - | MTHFD1L | Intron Variant |
|  | rs9455973 | chr6:168182326 | - | - | None | None |
|  | rs6942930 | chr7:1512784 | - | - | None | None |
|  | rs2039461 | chr9:20145990 | - | - | SLC24A2 | Intron Variant |
|  | rs7893928 | chr10:44583495 | - | - | LOC124902533 | Intron Variant |
|  | rs11610206 | chr12:47245743 | - | - | LOC105369746 | Intron Variant |
|  | rs2387100 | chr13:27852622 | - | - | PLUT | Intron Variant |
|  | rs9544105 | chr13:75984017 | - | - | None | None |
|  | rs659628 | chr13:76889101 | - | - | None | None |
|  | rs12146962 | chr14:32911892 | - | - | None | None |
|  | rs4555132 | chr15:97396008 | - | - | LINC02253, LINC02254 | Intron Variant, Intron Variant |
|  | rs1480090 | chr15:98172432 | - | - | None | None |
|  | rs1383139 | chr15:98174448 | - | - | None | None |
|  | rs1402627 | chr18:4133739 | - | - | DLGAP1 | Intron Variant |
|  | rs4459653 | chr19:44095462 | - | - | ZNF224 | Intron Variant |
|  | rs4802207 | chr19:44096224 | - | - | ZNF224 | Intron Variant |
|  | rs3746319 | chr19:44108078 | - | - | ZNF224, ZNF225-AS1 | Missense Variant, Non Coding Transcript Variant |
|  | rs2061332 | chr19:44109508 | - | - | ZNF225-AS1, ZNF224, ZNF225 | Intron Variant, 3 Prime UTR Variant, 2KB Upstream Variant |
|  | rs6059244 | chr20:31422680 | - | - | DEFB122 | Intron Variant |
|  | rs2180566 | chr20:31431051 | - | - | DEFB122 | 2KB Upstream Variant |
|  |  |  |  |  |  |  |
| Chia et al, 2021 | rs2230288 | chr1:155236376 | T | + | GBA | Missense Variant |
|  | rs6733839 | chr2:127135234 | T | + | LOC105373605 | Intron Variant |
|  | rs6599388 | chr4:945299 | T | + | TMEM175 | Intron Variant |
|  | rs7680557 | chr4:89842209 | C | - | SNCA-AS1 | 500B Downstream Variant |
|  | rs769449 | chr19:44906745 | A | + | APOE | Intron Variant |
|  |  |  |  |  |  |  |
| Schrijvers et al, 2012 | rs12007229 | chrX:67528513 | A | + | None | None |
|  | rs10491487 | chr5:81027549 | C | + | RASGRF2 | Intron Variant |
|  | rs2497931 | chrX:67436434 | C | + | None | None |
|  | rs4485213 | chr13:105256524 | A | + | None | None |
|  | rs26906 | chr5:81069068 | A | + | RASGRF2 | Intron Variant |
|  | rs17195948 | chr3:179738008 | A | + | USP13 | Intron Variant |
|  | rs4800982 | chr18:55142172 | A | + | None | None |
|  | rs484304 | chr13:50604234 | G | + | LOC107984567 | Genic Upstream Transcript Variant |
|  | rs2497944 | chrX:67347900 | A | + | None | None |
|  | rs5965383 | chrX:67299666 | C | + | None | None |
|  | rs721451 | chrX:67356482 | G | + | None | None |
|  | rs12396249 | chrX:67557224 | A | + | AR | Intron Variant |
|  | rs589080 | chr5:81930729 | C | + | None | None |
|  | rs2497938 | chrX:67343176 | G | + | None | None |
|  | rs2207080 | chrX:67353901 | G | + | None | None |
|  | rs2335508 | chrX:67300808 | A | + | None | None |
|  | rs2223842 | chrX:67344510 | C | + | None | None |
|  | rs2473895 | chrX:67351195 | A | + | None | None |
|  | rs2497928 | chrX:67428374 | C | + | None | None |
|  | rs2336175 | chrX:67320269 | A | + | None | None |
|  | rs2497939 | chrX:67344172 | C | + | None | None |
|  | rs2223841 | chrX:67350329 | G | + | None | None |
|  | rs2473896 | chrX:67350885 | G | + | None | None |
|  | rs2335506 | chrX:67299872 | G | + | None | None |
|  | rs2473897 | chrX:67346608 | G | + | None | None |
|  | rs2497943 | chrX:67346254 | C | + | None | None |
|  | rs5919363 | chrX:67292261 | G | + | None | None |
|  | rs2473891 | chrX:67356631 | A | + | None | None |
|  | rs6625174 | chrX:67310690 | G | + | None | None |
|  | rs687543 | chr5:81924368 | A | + | None | None |
|  | rs2878642 | chrX:67318887 | A | + | None | None |
|  | rs2473849 | chrX:67444241 | A | + | None | None |
|  | rs5919362 | chrX:67288837 | A | + | None | None |
|  | rs5919393 | chrX:67605515 | G | + | AR | Intron Variant |
|  | rs4827539 | chrX:67287254 | G | + | None | None |
|  | rs6625163 | chrX:67291142 | G | + | None | None |
|  | rs4601479 | chrX:67290616 | G | + | None | None |
|  | rs2497911 | chrX:67407598 | A | + | None | None |
|  | rs1024852 | chr3:179764694 | G | + | USP13 | Intron Variant |
|  | rs12014709 | chrX:67718624 | C | + | AR | Intron Variant |
|  | rs7868152 | chr9:130241664 | A | + | MIR12126 | 500B Downstream Variant |
|  | rs2540525 | chr13:50680576 | A | + | LOC107984567 | Genic Upstream Transcript Variant |
|  | rs9636785 | chr21:26760062 | G | + | None | None |
|  | rs10010358 | chr4:5404078 | A | + | STK32B | Intron Variant |
|  | rs16916777 | chr9:98518893 | A | + | GABBR2 | Intron Variant |
|  | rs6930720 | chr6:8170873 | A | + | LOC105374910 | Intron Variant |
|  | rs3015361 | chr13:105249152 | C | + | None | None |
|  | rs17738042 | chr10:103596274 | A | + | SH3PXD2A | 3 Prime UTR Variant |
|  | rs11632498 | chr15:58240308 | A | + | LOC124903499 | Intron Variant |
|  | rs17110927 | chr12:72208265 | G | + | TRHDE | Intron Variant |
|  | rs1950268 | chr14:97041951 | A | + | LOC105370647 | Intron Variant |
|  | rs9365597 | chr6:163685707 | G | + | None | None |
|  | rs7191284 | chr16:25317874 | C | + | None | None |
|  | rs2045262 | chr18:67375848 | G | + | None | None |
|  | rs11661371 | chr18:5396977 | A | + | EPB41L3 | Intron Variant |
|  | rs10899949 | chr10:43910757 | G | + | LINC00841, LINC02659 | Intron Variant, Intron Variant |
|  | rs2395148 | chr6:32353777 | A | + | TSBP1, TSBP1-AS1 | Intron Variant, Intron Variant |
|  | rs10839548 | chr11:6301054 | A | + | None | None |
|  | rs7989487 | chr13:105276911 | G | + | None | None |
|  | rs1453342 | chr11:77357725 | A | + | PAK1 | Intron Variant |
|  | rs7717830 | chr5:80983179 | A | + | RASGRF2 | Intron Variant |
|  | rs299541 | chr2:123068986 | G | + | LINC01826 | Intron Variant |
|  | rs6624304 | chrX:67655914 | A | + | AR | Intron Variant |
|  | rs6036158 | chr20:22600422 | G | + | LNCNEF | Intron Variant |
|  | rs11253424 | chr10:767486 | A | + | None | None |
|  | rs13040003 | chr20:61065674 | A | + | None | None |
|  | rs1510787 | chr4:24285296 | G | + | PPARGC1A | Intron Variant |
|  | rs12139692 | chr1:71995699 | A | + | NEGR1 | Intron Variant |
|  | rs1203917 | chr20:22595877 | G | + | LNCNEF | Intron Variant |
|  | rs797516 | chr13:50656485 | G | + | LOC107984567 | Genic Upstream Transcript Variant |
|  | rs4076597 | chr15:97397580 | G | + | LINC02253, LINC02254 | Intron Variant, Intron Variant |
|  | rs11238781 | chr10:43933537 | G | + | LINC00841 | Intron Variant |
|  | rs17039713 | chr2:60109454 | A | + | None | None |
|  | rs797512 | chr13:50652818 | G | + | LOC107984567 | Genic Upstream Transcript Variant |
|  | rs4827556 | chrX:67842648 | G | + | None | None |
|  | rs1011526 | chrX:66196245 | A | + | HEPH | Intron Variant |
|  | rs5919432 | chrX:67801708 | G | + | None | None |
|  | rs10899952 | chr10:43931573 | A | + | LINC00841 | Intron Variant |
|  | rs203467 | chr17:19906436 | A | + | AKAP10 | Intron Variant |
|  | rs4456006 | chrX:67808380 | C | + | None | None |
|  | rs7054364 | chrX:66172794 | A | + | HEPH | Intron Variant |
|  | rs13436118 | chr5:135069471 | A | + | PITX1-AS1 | Intron Variant |
|  | rs11776337 | chr8:5585547 | A | + | None | None |
|  | rs4420312 | chr12:130845628 | A | + | None | None |
|  | rs663519 | chr1:100127097 | G | + | SASS6 | Intron Variant |
|  | rs11062164 | chr12:2224486 | A | + | CACNA1C, CACNA1C-AS4 | Intron Variant, 2KB Upstream Variant |
|  | rs9608216 | chr22:23915592 | A | + | None | None |
|  | rs9972386 | chr15:82161942 | G | + | EFL1 | Intron Variant |
|  | rs10935154 | chr3:135111690 | A | + | EPHB1 | Intron Variant |
|  | rs797515 | chr13:50653279 | A | + | LOC107984567 | Genic Upstream Transcript Variant |
|  | rs17050873 | chr4:139709983 | A | + | MGST2 | Intron Variant |
|  | rs3886594 | chr18:67556556 | A | + | DSEL-AS1 | Intron Variant |
|  | rs8113515 | chr19:43311416 | C | + | None | None |
|  | rs989345 | chrX:67081192 | G | + | None | None |
|  | rs195113 | chr4:5411798 | G | + | STK32B | Intron Variant |
|  | rs11706256 | chr3:135122610 | G | + | EPHB1 | Intron Variant |
|  | rs532649 | chrX:67047406 | G | + | None | None |
|  | rs10486820 | chr7:81318201 | G | + | None | None |
|  | rs1931109 | chr9:85119605 | C | + | LOC107987088 | Intron Variant |
|  | rs485454 | chrX:67048360 | G | + | None | None |
|  | rs7572928 | chr2:234375711 | A | + | None | None |
|  | rs10183651 | chr2:123100166 | A | + | None | None |
|  | rs938059 | chrX:67116885 | C | + | None | None |
|  | rs17088268 | chr9:85144578 | A | + | LOC107987088 | 2KB Upstream Variant |
|  | rs12357879 | chr10:88738364 | A | + | LIPK | Intron Variant |
|  | rs141127 | chr6:84779516 | G | + | LOC124901494 | Intron Variant |
|  | rs5918694 | chrX:67098926 | A | + | None | None |
|  | rs1939469 | chr11:76525176 | G | + | EMSY | Intron Variant |
|  | rs1511060 | chrX:67096589 | G | + | None | None |
|  | rs12348840 | chr9:85124822 | A | + | LOC107987088 | Intron Variant |
|  | rs1988995 | chrX:67127842 | A | + | None | None |
|  | rs7602743 | chr2:156673142 | A | + | LINC01958 | Intron Variant |
|  | rs4827392 | chrX:67102120 | C | + | None | None |
|  | rs11060130 | chr12:129097311 | C | + | TMEM132D | Intron Variant |
|  | rs17056109 | chr5:158710431 | A | + | EBF1 | Intron Variant |
|  |  |  |  |  |  |  |
| Reus et al, 2021 | rs76679949 | chr5:168793635 | C | + | SLIT3 | Intron Variant |
|  | rs147211831 | chr9:27436086 | A | + | MOB3B | Intron Variant |
|  | rs117204439 | chr9:27607975 | C | + | None | None |
|  |  |  |  |  |  |  |
| Ferrari et al, 2015 | rs906175 | chr17:81199662 | T | + | CEP131 | Intron Variant |
|  | rs17042852 | chr2:52372929 | C | + | LINC01867, LOC730100 | Intron Variant, Intron Variant |
|  | rs1526678 | chr2:52408589 | G | + | None | None |
|  | rs17042770 | chr2:52344255 | C | + | LOC730100 | Intron Variant |
|  | rs2725391 | chr17:81218630 | T | + | CEP131 | Intron Variant |
|  | rs12621157 | chr2:52282738 | T | + | LOC730100 | Intron Variant |
|  | rs12622570 | chr2:52319163 | C | + | LOC730100 | Intron Variant |
|  | rs969413 | chr17:81222014 | A | + | CEP131 | Intron Variant |
|  | rs2659030 | chr17:81204174 | A | + | CEP131 | Intron Variant |
|  | rs2255166 | chr17:81239762 | C | + | NDUFAF8, TEPSIN | Intron Variant, 2KB Upstream Variant |
|  | rs12619513 | chr2:52305736 | A | + | LOC730100 | Intron Variant |
|  | rs9319617 | chr17:81218646 | C | - | CEP131 | Intron Variant |
|  | rs1048775 | chr17:81228529 | G | - | TEPSIN, LOC105371925 | 3 Prime UTR Variant, 2KB Upstream Variant |
|  | rs12614311 | chr2:52294578 | T | + | LOC730100 | Intron Variant |
|  |  |  |  |  |  |  |
| Ferrari et al, 2014 | rs302652 | chr11:88161663 | A | - | RAB38 | Intron Variant |
|  | rs74977128 | chr11:88203706 | C | + | None | None |
|  | rs9268877 | chr6:32463370 | A | + | None | None |
|  | rs9268856 | chr6:32461942 | A | - | None | None |
|  | rs1980493 | chr6:32395438 | C | - | TSBP1-AS1, BTNL2 | Intron Variant, Intron Variant |

**Supplementary Figure 1**

**
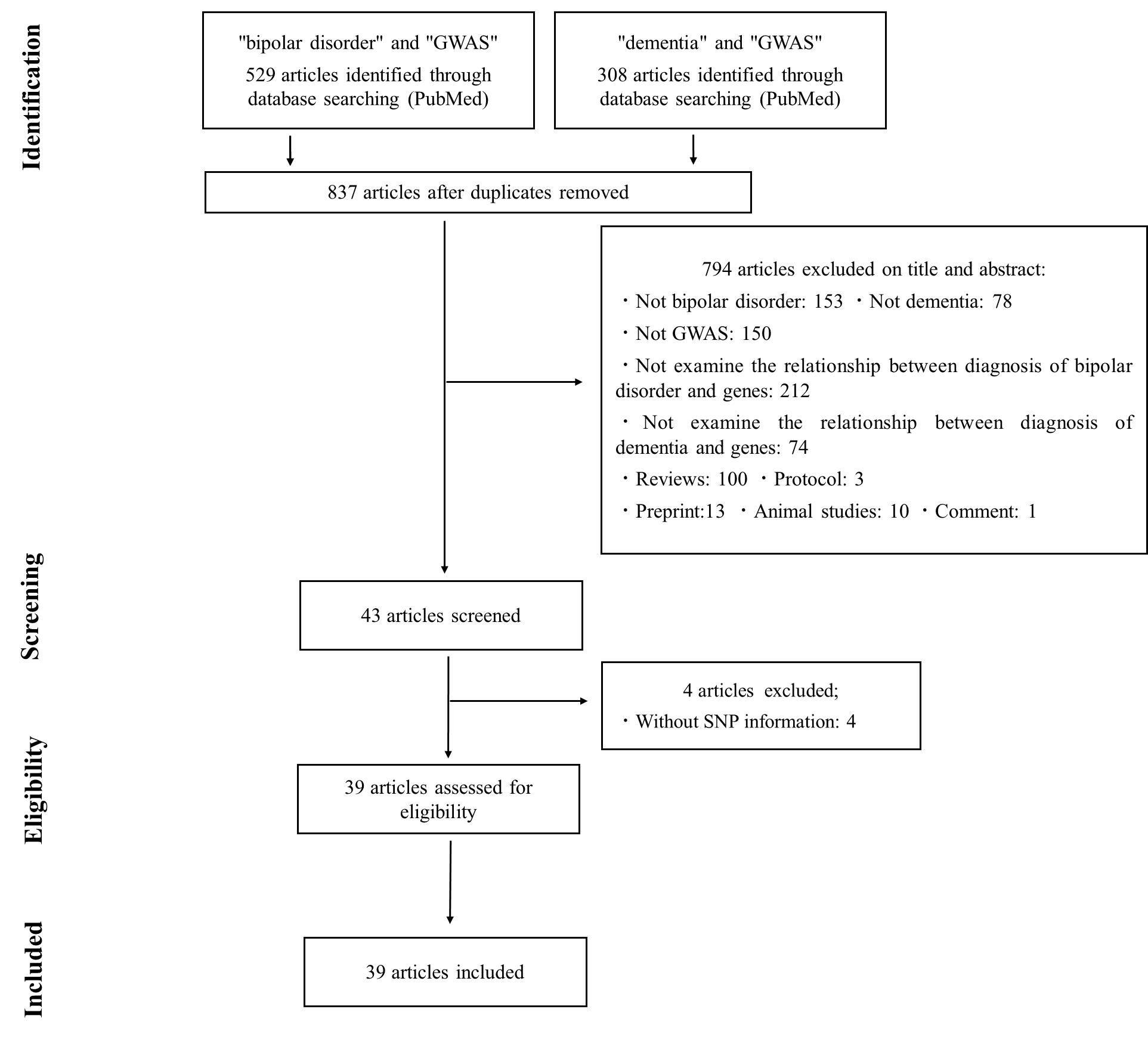
**

**Supplementary Figure 2**

**
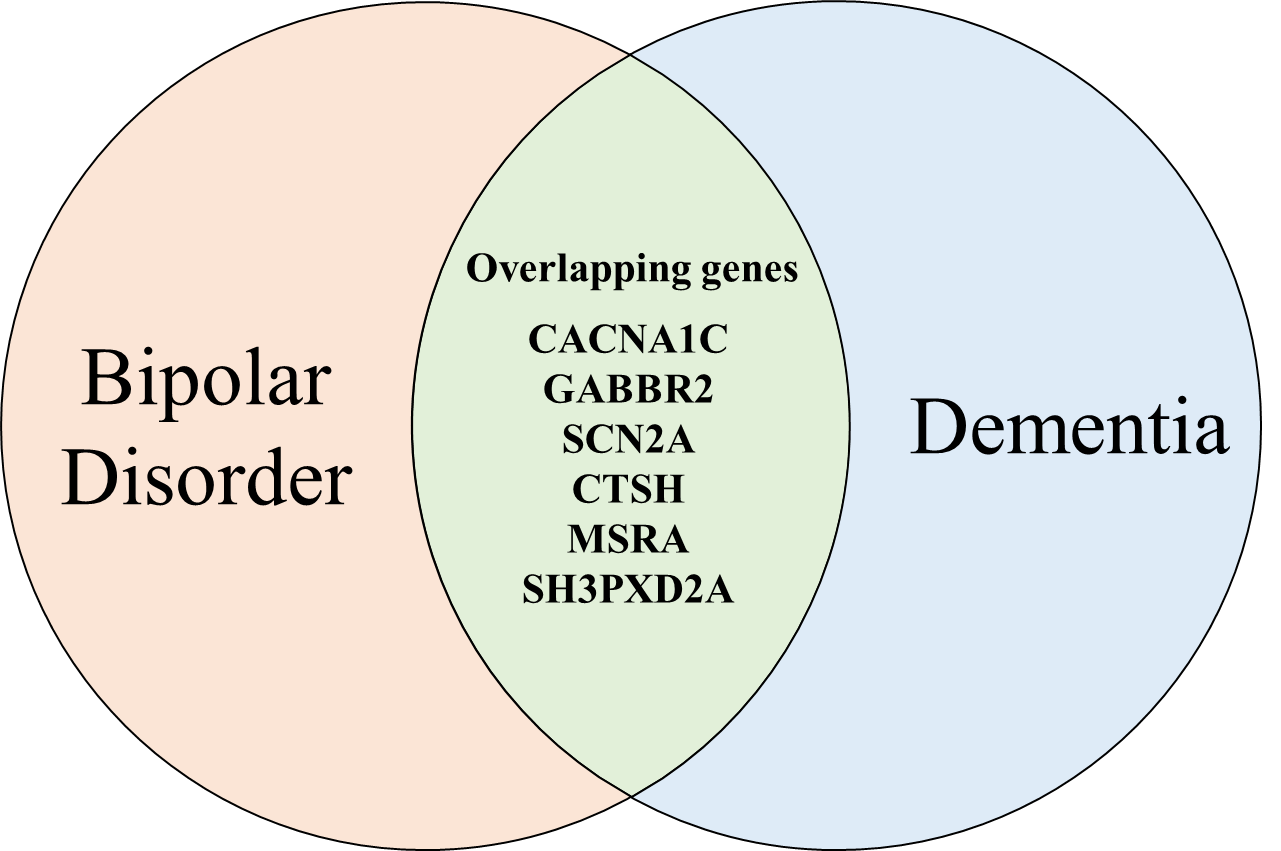
**

**Figure legend**

**Supplementary Figure 1. Flow chart of the study**

Initially, 837 article were found using the search terms. Finally, we included 39 articles for this review.

**Supplementary Figure 2. The overlapping genes with bipolar disorder and cognition**

Green area is the overlapping genes with bipolar disorder and dementia.
